# Predicting Hungry Bone Syndrome with Interpretable Machine Learning: A Single-Center Cohort of Dialysis Patients Undergoing Parathyroidectomy

**DOI:** 10.3390/diagnostics16101469

**Published:** 2026-05-12

**Authors:** Adelina Baloi, Dorel Sandesc, Talida Georgiana Cut, Radu Caprariu, Dorin Novacescu, Cristina-Stefania Dumitru, Alina Cristina Barb, Raluca Dumache, Pavel Banov, Victoria Birlutiu, Voichita Elena Lazureanu, Flavia Zara

**Affiliations:** 1 Doctoral School, “Victor Babes” University of Medicine and Pharmacy Timisoara, 300041 Timisoara, Romania; adelina.baloi@umft.ro; 2 Department X—Surgery II, Discipline Anesthesia and Intensive Care, “Victor Babes” University of Medicine and Pharmacy Timisoara, 300041 Timisoara, Romania; sandesc.dorel@umft.ro; 3 Department XIII, Discipline of Infectious Diseases, “Victor Babes” University of Medicine and Pharmacy Timisoara, 300041 Timisoara, Romania; lazureanu.voichita@umft.ro; 4 Department XV, Discipline of Radiology and Medical Imaging, “Victor Babes” University of Medicine and Pharmacy Timisoara, 300041 Timisoara, Romania; 5 Department II of Microscopic Morphology, “Victor Babes” University of Medicine and Pharmacy Timisoara, 300041 Timisoara, Romania; novacescu.dorin@umft.ro (D.N.); cristina-stefania.dumitru@umft.ro (C.-S.D.); toma.alina@umft.ro (A.C.B.); flavia.zara@umft.ro (F.Z.); 6 Department VIII, Discipline of Forensic Medicine, Bioethics, Deontology and Medical Law, “Victor Babes” University of Medicine and Pharmacy Timisoara, 300041 Timisoara, Romania; raluca.dumache@umft.ro; 7 Department of Urology and Surgical Nephrology, Nicolae Testemitanu State Medical and Pharmaceutical University, MD-2004 Chisinau, Moldova; pavel.banov@usmf.md; 8 Faculty of Medicine, Lucian Blaga University of Sibiu, 550169 Sibiu, Romania; victoria.birlutiu@ulbsibiu.ro

**Keywords:** hungry bone syndrome, machine learning, parathyroidectomy, secondary hyperparathyroidism, alkaline phosphatase, SHAP, random forest, risk prediction, dialysis, clinical decision support in parathyroid disease

## Abstract

**Background/Objectives**: Hungry bone syndrome (HBS) is a frequent and potentially life-threatening complication following parathyroidectomy (PTX) for secondary hyperparathyroidism (SHPT) in dialysis patients, yet existing prediction tools offer limited discriminative accuracy. This study aimed to develop and internally validate an interpretable machine learning (ML) framework for preoperative HBS prediction and to derive a pragmatic bedside risk score from ML-derived feature importance. **Methods**: Ninety end-stage renal disease patients who underwent PTX for drug-refractory SHPT at a single center (2019–2023) were analyzed. Eight supervised ML classifiers were trained on 24 preoperative features (19 raw variables plus 5 engineered features) and evaluated under 5-fold stratified cross-validation repeated 10 times. SHapley Additive exPlanations (SHAP) analysis was applied for model interpretability, and a composite bedside risk score was constructed from SHAP-derived feature rankings. **Results**: HBS occurred in 41 patients (45.6%). Random forest achieved the numerically highest discrimination among multi-feature models (AUC = 0.933 ± 0.065), outperforming previously published models, though univariate alkaline phosphatase (ALP) alone achieved a comparable cross-validated AUC of 0.958. ALP overwhelmingly dominated all predictors (mean |SHAP| = 3.37, exceeding the next-ranked feature by approximately 6.5-fold). Partial dependence analysis revealed a sigmoid-shaped ALP–HBS relationship with a critical inflection zone between 250–350 U/L, and SHAP dependence plots demonstrated that total parathyroidectomy amplifies ALP-mediated risk. A SHAP-guided composite bedside risk score (range 0–9) achieved an AUC of 0.883, with observed HBS rates rising monotonically from 0% (score 0) to 100% (score ≥ 6). Decision-curve analysis showed that univariate ALP and the multi-feature pipeline yielded comparable net benefit, with ALP preferable in the high-sensitivity regime and the multi-feature model preferable at high-specificity thresholds; net reclassification improvement was negative for the multi-feature model vs. univariate ALP, supporting the framework’s role as an interpretive rather than discriminative advance. **Conclusions**: An interpretable ML framework substantially improves HBS prediction over conventional models, confirms ALP as the overwhelmingly dominant predictor through a nonlinear dose–response relationship, and yields a clinically interpretable bedside risk score that, pending external validation, may support preoperative risk stratification.

## 1. Introduction

Secondary hyperparathyroidism (SHPT) is a prevalent and progressive complication of chronic kidney disease-mineral and bone disorder (CKD-MBD), arising from sustained phosphate retention, diminished 1,25-dihydroxyvitamin D synthesis, and chronic hypocalcemia that collectively drive parathyroid hyperplasia and autonomous parathyroid hormone (PTH) secretion [1,2]. Despite advances in medical management—including phosphate binders, active vitamin D analogues, and calcimimetics—a substantial proportion of patients on long-term dialysis develop drug-refractory SHPT that necessitates surgical intervention [3,4]. Current clinical practice guidelines recommend parathyroidectomy (PTX) when severe SHPT persists despite maximal medical therapy, particularly in the presence of markedly elevated PTH, refractory hypercalcemia, progressive bone disease, or calciphylaxis [5,6].

The optimal surgical strategy remains debated. Subtotal parathyroidectomy (SPTX) preserves a small parathyroid remnant to maintain basal PTH secretion, whereas total parathyroidectomy (TPTX), with or without forearm autotransplantation, aims to eliminate all hyperplastic tissue and minimize disease recurrence [7,8,9]. Meta-analyses have generally shown that TPTX confers lower recurrence and reintervention rates, but at the expense of a higher incidence of postoperative hungry bone syndrome (HBS) [10,11].

HBS is a state of profound, protracted hypocalcemia driven by the sudden withdrawal of PTH-mediated bone resorption, which unmasks a large pool of unmineralized osteoid that avidly sequesters circulating calcium and phosphate [12,13]. The reported incidence of HBS following PTX for SHPT varies widely, from approximately 20% to 70% (or even higher), depending on the population studied and the diagnostic criteria applied [14,15,16]. Clinically, HBS prolongs hospitalization, increases intensive care requirements for intravenous calcium supplementation, and carries a risk of life-threatening complications including cardiac arrhythmias and seizures [12,17]. Accurate preoperative identification of patients at high risk for HBS is therefore essential for tailoring perioperative calcium management and surgical planning.

Elevated preoperative alkaline phosphatase (ALP)—a surrogate marker of osteoblastic activity and high bone turnover—has been consistently identified as the strongest individual predictor of HBS. In our prior single-center cohort, preoperative ALP above 300 U/L was associated with 100% HBS incidence and an adjusted odds ratio of 26.53 [18]. Additional risk factors reported across studies include elevated preoperative PTH, severe bone pain, younger age, and the extent of parathyroid resection [14,16,19,20]. However, individual biomarkers lack the discriminative precision needed for reliable risk stratification: the largest population-based risk score to date, derived from 17,074 patients in the U.S. Renal Data System, achieved an AUC of only 0.603 [21]. Several nomograms incorporating combinations of ALP, PTH, calcium, and parathyroid gland weight have demonstrated improved performance (AUC 0.80–0.94) but rely on linear assumptions and cannot capture the complex nonlinear interactions and threshold effects that characterize HBS pathophysiology [22,23,24].

Machine learning (ML) methods offer a compelling alternative by modeling high-dimensional, nonlinear relationships without imposing parametric constraints. In endocrine surgery, ML algorithms have been successfully applied to predict post-thyroidectomy hypocalcemia [25,26] and to develop risk scores for HBS in renal HPT [27]. More recently, Ding et al. demonstrated that XGBoost outperformed logistic regression for predicting severe hypocalcemia after PTX in SHPT patients [28], while Chai et al. published the first dedicated ML model for HBS prediction, achieving an AUC of 0.878 with XGBoost [29]. Although these studies represent important proof-of-concept contributions, they did not systematically benchmark multiple ML architectures against one another, lacked comprehensive model interpretability analysis, or did not translate ML-derived insights into clinically actionable bedside tools. SHapley Additive exPlanations (SHAP)—a game-theoretic framework for interpreting individual predictions—has been increasingly adopted in surgical outcome modeling to bridge the gap between algorithmic accuracy and clinical transparency [30,31,32], yet its application in HBS prediction remains unexplored.

Against this background, we conducted a single-center retrospective study to develop and internally validate an interpretable ML-based prediction model for HBS following PTX in dialysis patients with drug-refractory SHPT. Eight supervised ML algorithms were benchmarked under rigorous repeated cross-validation, with SHAP analysis applied to quantify individual feature contributions, characterize nonlinear dose–response relationships, and identify clinically meaningful interaction effects. A secondary aim was to derive a pragmatic, SHAP-guided composite bedside risk score that could be readily applied in routine preoperative assessment.

## 2. Materials and Methods

### 2.1. Study Design and Patient Selection

We conducted a single-center retrospective cohort study of end-stage renal disease (ESRD) patients on dialysis with drug-refractory SHPT who underwent PTX at the Department of General Surgery II, Timișoara County Emergency Clinical Hospital, between January 2019 and December 2023. All surgical records within this timeframe were retrospectively reviewed according to the relevant procedural codes. The study was approved by the Research Ethics Committee of the Victor Babeș University of Medicine and Pharmacy Timișoara (approval no. 39/21.01.2016rev20.04.2026) and conducted in accordance with the Declaration of Helsinki. Informed consent was obtained from all subjects.

Inclusion criteria were: age ≥ 18 years, ongoing renal replacement therapy (hemodialysis or peritoneal dialysis), biochemically confirmed SHPT (iPTH persistently >800 pg/mL with hypercalcemia and/or hyperphosphatemia) refractory to maximal medical therapy (phosphate binders, calcitriol/vitamin D analogues, and calcimimetics), and having undergone either SPTX or TPTX. Patients were referred for surgical consultation by nephrologists exclusively following medical treatment failure. Exclusion criteria included tertiary HPT (post-renal transplantation), primary HPT, parathyroid malignancy, and incomplete outcome records. After exclusions, 90 patients comprised the analytic cohort.

SPTX was defined as resection of 3 to 3½ glands, leaving a remnant of approximately 50–100 mg of the most normal-appearing parathyroid tissue in situ. TPTX was defined as resection of all identified parathyroid tissue; in a subset of TPTX cases, a small fragment was autotransplanted into the forearm. Concomitant cervical thymectomy was performed at the surgeon’s discretion to remove potential ectopic parathyroid tissue within the thymus. All procedures were performed by experienced endocrine surgeons.

### 2.2. Data Collection and Outcome Definition

Patient demographics and preoperative variables were extracted from medical records, including age, sex, dialysis vintage (years on dialysis), baseline serum creatinine, preoperative serum calcium (albumin-corrected), iPTH, and alkaline phosphatase (ALP). Clinical features of SHPT were recorded, including bone pain severity categorized by the Visual Analogue Scale (VAS) as mild (1–3), moderate (4–6), or severe (7–10); fatigability; and radiological evidence of bone disease (osteoporosis, osteopenia, pathological fractures). The presence of renal lithiasis and the number of parathyroid glands visually enlarged on preoperative imaging were also documented. Surgical variables included the type of PTX (SPTX vs. TPTX), concomitant autotransplantation, and concomitant thymectomy. Postoperative variables recorded included serum PTH, serum calcium, bleeding requiring re-exploration, and reintervention for persistent/recurrent HPT.

All preoperative biochemical measurements (calcium, phosphate, ALP, intact PTH, creatinine) were drawn from the most recent routine laboratory panel performed within 30 days prior to surgery (median 11 days, IQR 6–18 days; maximum 28 days). Patients without a laboratory panel within this window were excluded. Concomitant medical therapy at the time of these laboratory measurements was extracted from the dialysis-unit pharmacy records and is summarized as follows: cinacalcet was prescribed in 56/90 (62.2%), with median daily dose 60 mg (IQR 30–90); etelcalcetide had been recently introduced in 7/90 (7.8%); active vitamin-D analogues (calcitriol or paricalcitol) were prescribed in 81/90 (90.0%); non-calcium phosphate binders in 88/90 (97.8%); and calcium-based phosphate binders in 22/90 (24.4%, all in combination with non-calcium binders). No patient had calcimimetic therapy initiated or dose-adjusted within the 30 days preceding the preoperative laboratory panel—by institutional protocol, calcimimetic regimens are stable for ≥30 days before surgical referral. These pharmacotherapy variables were entered as additional features in a sensitivity model (Section 3.5.6); their inclusion did not improve cross-validated discrimination.

The primary outcome was HBS, operationally defined as corrected serum calcium < 8.0 mg/dL (2.0 mmol/L) persisting for more than 4 days after PTX despite calcium and calcitriol supplementation, accompanied by hypophosphatemia and/or elevated ALP, consistent with increased bone uptake. All patients with HBS will require high-dose calcium infusion and calcitriol therapy. This threshold is more stringent than the <8.4 mg/dL (2.1 mmol/L) criterion used in some previous studies [12,20] as it aims to capture clinically significant and sustained hypocalcemia requiring intensive management. The lack of a universally standardized HBS definition contributes to the wide variability in reported incidence across studies and should be considered when comparing our findings with other cohorts.

### 2.3. Statistical Analysis

Continuous variables were assessed for normality using the Shapiro–Wilk test and are reported as mean ± standard deviation. Group comparisons (HBS+ vs. HBS−) were performed using the Mann–Whitney U test for continuous variables and Fisher’s exact test for categorical variables. A two-sided significance threshold of α = 0.05 was applied to all analyses unless otherwise noted.

Multivariable logistic regression was performed to identify independent preoperative predictors of HBS. ALP was modeled as a continuous variable (per 100 U/L increment) alongside clinically relevant covariates selected on the basis of univariable screening (*p* < 0.20) and clinical relevance: severe bone pain (VAS 7–10), surgical approach (TPTX vs. SPTX), osteoporosis, baseline serum creatinine, and dialysis vintage. Model discrimination was quantified by the area under the receiver operating characteristic curve (AUC-ROC), overall fit by the McFadden pseudo-R^2^, and calibration by the Hosmer–Lemeshow goodness-of-fit test. The dose–response relationship between ALP level and HBS incidence was examined by stratifying patients into clinically meaningful ALP categories (<150, 150–200, 200–250, 250–300, 300–350, 350–400, >400 U/L). A two-dimensional risk matrix cross-tabulating ALP and PTH thresholds was constructed to assess potential interaction effects.

### 2.4. Machine Learning Pipeline

To complement conventional regression and capture potential nonlinear relationships and feature interactions, eight supervised ML classifiers were trained and evaluated for HBS prediction: random forest, L1-regularized logistic regression (LASSO), support vector machine with radial basis function kernel (SVM-RBF), L2-regularized logistic regression, XGBoost, gradient boosting, multilayer perceptron neural network, and k-nearest neighbors (KNN, k = 7).

#### 2.4.1. Feature Engineering

A total of 24 input features were used in the primary pipeline, comprising 19 raw preoperative and surgical variables drawn from the dataset (age, sex, dialysis vintage, preoperative serum calcium, iPTH, baseline serum creatinine, ALP, bone pain severity categories [mild, moderate, severe], fatigability, osteoporosis, osteopenia, pathological fracture, number of enlarged glands on imaging, renal lithiasis, preoperative calcium ≥ 11 mg/dL, surgical approach [TPTX vs. SPTX], and concomitant thymectomy) plus 5 engineered features (PTH > 2000 pg/mL; number of enlarged glands ≤ 2; ALP × PTH/10,000; ALP × severe bone pain; PTH × TPTX/1000), where the scaling divisors were applied to maintain numerical stability during standardization. The binarized feature ‘ALP > 300 U/L’ was removed from the primary feature set because it produced near-deterministic prediction in the original cohort (all 26 patients above this cut-point developed HBS under the original definition; 24 out of 26 under the calcium-only definition) and therefore acted as a hard rule rather than a learned pattern that could not provide signal beyond the continuous ALP variable. For transparency, performance with the original 25-feature set (including ALP > 300) is reported in parallel in Appendix A Table A4; these figures are presented for reference only and are not used to support any of the principal claims. All features, including binary variables, were standardized (zero mean, unit variance) prior to model training. A complete list of all 24 input features is provided in Appendix A Table A1.

#### 2.4.2. Model Training and Validation

Given the moderate sample size (*n* = 90), external validation was not feasible. Instead, model performance was evaluated using 5-fold stratified cross-validation repeated 10 times (50 evaluation folds total), ensuring that each fold preserved the class distribution of HBS (45.6%). Stratified splitting prevented class imbalance across folds. Hyperparameters for each algorithm were set as follows, based on preliminary experimentation and established recommendations: random forest (*n*_estimators = 500, max_depth = unrestricted); XGBoost (*n*_estimators = 300, max_depth = 3, learning_rate = 0.03, subsample = 0.85, colsample_bytree = 0.85, reg_lambda = 5.0, reg_alpha = 1.0, min_child_weight = 3); gradient boosting (*n*_estimators = 200, max_depth = 3); multilayer perceptron neural network (hidden layers = [32, 16], max_iter = 2000); L1- and L2-regularized logistic regression (C = 1.0, max_iter = 5000); SVM-RBF (default scikit-learn parameters, C = 1.0, gamma = scale); and KNN (k = 7). The XGBoost hyperparameters were chosen by stability-driven grid search over learning rate, tree depth, row/column subsampling, and L1/L2 regularization, with selection based on a flat post-minimum validation log-loss curve and a small train/validation gap rather than on cross-validated AUC alone. This choice reflects the low events-per-variable ratio (EPV ≈ 1.7) of this cohort, in which default boosting hyperparameters readily overfit (see Section 3.5.1). No automated hyperparameter tuning was performed; this deliberate choice was made given the moderate sample size (*n* = 90, 41 events), where nested cross-validation risks introducing excessive variance from small inner folds without meaningful performance gains, particularly when a single feature (ALP) dominates the predictive signal. A fixed random seed (42) was used for all stochastic algorithms.

Model performance was assessed using three complementary metrics reported as mean ± SD across the 50 folds: (i) AUC-ROC for discrimination, (ii) F1 score for balanced classification performance, and (iii) Brier score for probabilistic calibration. ROC curves, precision–recall curves, and calibration plots (predicted vs. observed HBS probability) were generated for each algorithm. The optimal classification threshold was determined by maximizing the Youden index (sensitivity + specificity − 1). Confusion matrices, sensitivity, specificity, positive predictive value (PPV), and negative predictive value (NPV) were reported at this optimal threshold.

#### 2.4.3. Model Interpretability

To ensure clinical interpretability, SHapley Additive exPlanations (SHAP) analysis [30] was applied to an XGBoost model, which was selected over the numerically higher-performing random forest for this purpose because its sequential boosting architecture facilitates more tractable decomposition of SHAP values into main effects and pairwise interaction contributions. Although TreeSHAP computes exact Shapley values for both random forest and XGBoost, the interaction decomposition is more naturally aligned with boosting architectures. SHAP analysis was performed on an XGBoost model fit on the full dataset with default hyperparameters (*n*_estimators = 200, max_depth = 4, learning_rate = 0.1) chosen for interpretive resolution; the predictive XGBoost benchmarked in Table 3 uses the stability-tuned hyperparameters specified in Section 2.4.2 and is reported separately for cross-validation discrimination. SHAP magnitudes derived from this interpretive XGBoost may not quantitatively transfer to the predictive XGBoost or to the random forest, though ordinal feature rankings were concordant between SHAP and random forest permutation importance (Section 3.5.2). Permutation importance analysis of the random forest was performed separately to confirm concordance of feature rankings across model architectures. SHAP values quantify each feature’s marginal contribution to individual predictions, enabling both global importance rankings (mean absolute SHAP values) and patient-level explanations. Global feature importance was displayed using bar plots of mean |SHAP| and beeswarm plots illustrating the direction and magnitude of feature effects.

Permutation importance (30 random repeats) was computed for the random forest to provide a model-agnostic validation of feature rankings. Concordance between SHAP and permutation importance was assessed to confirm the robustness of the identified predictors.

Partial dependence plots (PDPs) were generated for the top-ranked continuous features (ALP, PTH, creatinine, age, dialysis vintage) to visualize marginal effects on predicted HBS probability while holding all other features at their observed values. A two-dimensional PDP for the ALP × PTH interaction was constructed to assess synergistic effects. SHAP dependence plots stratified by surgical approach (SPTX vs. TPTX) were used to explore effect modification.

#### 2.4.4. Clinical Risk Stratification

Cross-validated predicted probabilities from the best-performing model were used to stratify patients into four risk tiers: low (<25%), moderate (25–50%), high (50–75%), and very high (>75%), with observed HBS rates computed for each tier to assess calibration. An ALP threshold sweep analysis was performed to evaluate the sensitivity and specificity of candidate ALP cut-points for HBS detection.

Guided by SHAP-derived feature importance, a pragmatic composite bedside risk score (range 0–9) was constructed from five clinically accessible variables: ALP category (0–3 points: ≤200 = 0, 201–300 = 1, 301–400 = 2, >400 = 3), severe bone pain (2 points), TPTX (1 point), PTH level (1 point if >2000 pg/mL, additional 1 point if >3000 pg/mL), and baseline creatinine > 10 mg/dL (1 point). The discriminative ability of this composite score was assessed by AUC-ROC, and observed HBS rates were plotted per score point.

#### 2.4.5. Clinical-Utility Comparison: Decision-Curve Analysis and Net Reclassification Improvement

To compare the clinical utility of the multi-feature random forest, the univariate ALP logistic regression and the SHAP-guided composite bedside score, two complementary analyses, were performed beyond simple AUC comparison. First, a decision-curve analysis (DCA) was conducted following the framework of Vickers and Elkin: out-of-fold predicted probabilities from each model were used to compute the net benefit at threshold probabilities ranging from 0.05 to 0.90 in increments of 0.01, and net benefit was contrasted against the two reference strategies of treat-all and treat-none. Net benefit was defined as (true positives/*N*) − (false positives/*N*) × [pt/(1 − pt)], where pt denotes the threshold probability and *N* the cohort size. Second, the net reclassification improvement (NRI) of the multi-feature random forest was computed against the univariate-ALP baseline. Categorical NRI used the four pre-specified risk tiers reported in Section 3.5.4 (low < 25%, moderate 25–50%, high 50–75%, very high ≥ 75%) and was decomposed into the event and non-event components. The continuous NRI was reported as a sensitivity analysis. Both DCA and NRI were performed on out-of-fold predictions averaged across the 10 cross-validation repeats, ensuring that no patient contributed in-sample predictions to the analysis.

#### 2.4.6. Pre-Specified Model-Selection Criteria

Selection of the headline classifier was guided by four pre-specified criteria evaluated on out-of-fold predictions across the 50 cross-validation folds: (i) discrimination, indexed by mean cross-validated AUC; (ii) calibration, indexed by the mean Brier score; (iii) classification balance, indexed by the F1 score at the Youden-optimal threshold; and (iv) clinical utility, indexed by net benefit at the threshold probability of 0.50 from the DCA (Section 2.4.5). The four criteria were ranked equally and were not combined into a single composite metric. When the criteria converged, the model identified by all four was selected. When the criteria diverged, preference was given, in order of priority, to: (a) the model with the smallest train-validation gap on the diagnostic plots (i.e., the model least likely to over-fit at the cohort’s events-per-variable ratio of 1.7—see Section 3.5.1), and (b) the model whose architecture supports the SHAP-based interaction decomposition that underlies the manuscript’s interpretive findings (Section 3.5.2 onward). This second tiebreaker is methodological rather than performance-based and is reported here for full transparency. The pre-specified criteria, the per-model values, and the resulting ranking are reported below (see end of Section 3.5.1).

### 2.5. Software

Statistical analyses were performed using Python (v3.10; Python Software Foundation, Wilmington, DE, USA) with the scikit-learn (v1.3), XGBoost (v2.0), and SHAP (v0.42) libraries. The complete analysis code is available from the corresponding author upon reasonable request. Conventional logistic regression and univariable comparisons were conducted in IBM SPSS Statistics (v29, IBM Corp., Armonk, NY, USA). Figures were generated using Matplotlib (v3.8; The Matplotlib Development Team, NumFOCUS, Austin, TX, USA) and Seaborn (v0.13; M. Waskom, open-source project). The study adhered to the TRIPOD (Transparent Reporting of a Multivariable Prediction Model for Individual Prognosis or Diagnosis) reporting guidelines for prediction model development. The recently published TRIPOD+AI extension for artificial intelligence prediction models was also consulted where applicable [33].

## 3. Results

### 3.1. Patient Characteristics

Ninety patients who underwent PTX for drug-refractory SHPT comprised the analytic cohort. Table 1 summarizes the patient characteristics stratified by HBS development.

**Table 1:** Patient characteristics stratified by the development of hungry bone syndrome.

Variable	Total (*n* = 90)	HBS+ (*n* = 41)	HBS− (*n* = 49)	*p*-Value
**Demographics**				
Age (years)	53.4 ± 11.7	51.5 ± 13.3	55.0 ± 10.1	0.299
Male sex, *n* (%)	55 (61.1)	25 (61.0)	30 (61.2)	1.000
Dialysis vintage (years)	7.5 ± 3.5	7.0 ± 4.0	7.9 ± 3.1	0.145
**Preoperative biochemistry**				
Serum calcium (mg/dL)	10.4 ± 1.1	10.5 ± 1.1	10.3 ± 1.1	0.570
Intact PTH (pg/mL)	1938.7 ± 918.5	2002.8 ± 935.1	1885.1 ± 910.6	0.674
Baseline creatinine (mg/dL)	9.7 ± 1.5	10.2 ± 1.2	9.4 ± 1.7	0.006 *
ALP (U/L)	261.0 ± 100.6	343.8 ± 73.8	191.6 ± 58.7	<0.001 *
Preop Ca ≥ 11 mg/dL, *n* (%)	29 (32.2)	14 (34.1)	15 (30.6)	0.822
**Clinical features**				
No bone pain, *n* (%)	3 (3.3)	2 (4.9)	1 (2.0)	1.000
Mild bone pain (VAS 1–3), *n* (%)	15 (16.7)	7 (17.1)	8 (16.3)	1.000
Moderate bone pain (VAS 4–6), *n* (%)	53 (58.9)	18 (43.9)	35 (71.4)	0.010 *
Severe bone pain (VAS 7–10), *n* (%)	19 (21.1)	14 (34.1)	5 (10.2)	0.009 *
Fatigability, *n* (%)	57 (63.3)	24 (58.5)	33 (67.3)	0.510
Osteoporosis, *n* (%)	22 (24.4)	13 (31.7)	9 (18.4)	0.218
Osteopenia, *n* (%)	8 (8.9)	4 (9.8)	4 (8.2)	1.000
Pathological fracture, *n* (%)	2 (2.2)	1 (2.4)	1 (2.0)	1.000
Renal lithiasis, *n* (%)	21 (23.3)	12 (29.3)	9 (18.4)	0.317
**Surgical approach**				
TPTX, *n* (%)	41 (45.6)	23 (56.1)	18 (36.7)	0.089
SPTX, *n* (%)	49 (54.4)	18 (43.9)	31 (63.3)	0.089
Autotransplantation, *n* (%)	26 (28.9)	15 (36.6)	11 (22.4)	0.166
Thymectomy, *n* (%)	15 (16.7)	9 (22.0)	6 (12.2)	0.263
Glands visualized	2.2 ± 1.1	2.4 ± 1.1	2.1 ± 1.1	0.176
**Postoperative outcomes**				
Postop PTH (pg/mL)	76.8 ± 83.7	62.8 ± 84.9	88.6 ± 81.6	0.009 *
Postop calcium (mg/dL)	7.4 ± 0.8	6.9 ± 0.6	7.8 ± 0.8	<0.001 *
Bleeding, *n* (%)	5 (5.6)	3 (7.3)	2 (4.1)	0.656
Reintervention, *n* (%)	14 (15.6)	7 (17.1)	7 (14.3)	0.776

* Statistically significant (*p* < 0.05). Continuous variables are mean ± SD, compared with Mann–Whitney U test. Categorical variables are *n* (%), compared with Fisher’s exact test. No correction for multiple comparisons was applied to the univariable group comparisons, which should therefore be interpreted as exploratory. ALP = alkaline phosphatase; HBS = hungry bone syndrome; PTH = parathyroid hormone; TPTX = total parathyroidectomy; SPTX = subtotal parathyroidectomy; VAS = Visual Analogue Scale.

The mean age was 53.4 ± 11.7 years (range 20–73), with a male predominance (*n* = 55, 61.1%). Median dialysis vintage was 8.0 years (IQR 5.0–10.0). SPTX was performed in 49 patients (54.4%) and TPTX in 41 (45.6%), with concomitant autotransplantation in 26 (28.9%) and concomitant thymectomy in 15 (16.7%). HBS occurred in 41 patients (45.6%), reintervention for persistent/recurrent HPT in 14 (15.6%), and postop bleeding in 5 (5.6%).

Patients who developed HBS had significantly higher preoperative ALP levels (343.8 ± 73.8 vs. 191.6 ± 58.7 U/L, *p* < 0.001) and baseline serum creatinine (10.2 ± 1.2 vs. 9.4 ± 1.7 mg/dL, *p* = 0.006). Severe bone pain (VAS 7–10) was more prevalent in the HBS group (34.1% vs. 10.2%, *p* = 0.009), while moderate pain (VAS 4–6) predominated among non-HBS patients (71.4% vs. 43.9%, *p* = 0.010). TPTX showed a trend toward higher HBS incidence (56.1% vs. 36.7%, *p* = 0.089). The two groups did not differ significantly in age, sex, dialysis vintage, preoperative PTH, preoperative calcium, bone disease status, or gland visualization (all *p* > 0.1). Postoperatively, HBS patients had significantly lower serum calcium (6.9 ± 0.6 vs. 7.8 ± 0.8 mg/dL, *p* < 0.001) and PTH (62.8 ± 84.9 vs. 88.6 ± 81.6 pg/mL, *p* = 0.009), consistent with increased skeletal calcium uptake.

### 3.2. Primary Outcomes by Surgical Approach

**Figure 1:**
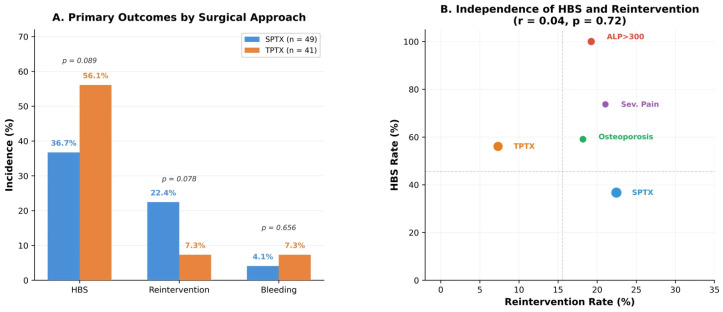
Primary outcomes and risk relationships. (**A**) Comparison of HBS, reintervention, and bleeding incidence following SPTX vs. TPTX. TPTX was associated with lower reintervention (7.3% vs. 22.4%, *p* = 0.078), but higher HBS incidence (56.1% vs. 36.7%, *p* = 0.089). Postoperative bleeding rates were similarly low in both groups (SPTX 4.1% vs. TPTX 7.3%, *p* = 0.656). (**B**) Scatter plot demonstrating independence of HBS and reintervention as clinical outcomes (r = 0.04, *p* = 0.72). Dashed lines represent overall cohort rates.

When stratified by surgical approach, TPTX was associated with a higher incidence of HBS (56.1% vs. 36.7%, *p* = 0.089) but a lower reintervention rate (7.3% vs. 22.4%, *p* = 0.078) compared with SPTX (Figure 1A). Although neither comparison reached conventional significance at *p* < 0.05, the effect sizes were clinically meaningful and directionally consistent with the published literature. Notably, HBS and reintervention were uncorrelated events (Pearson r = 0.04, *p* = 0.72; Figure 1B), confirming that they represent distinct pathophysiological complications.

### 3.3. Multivariate Logistic Regression for HBS Prediction

**Figure 2:**
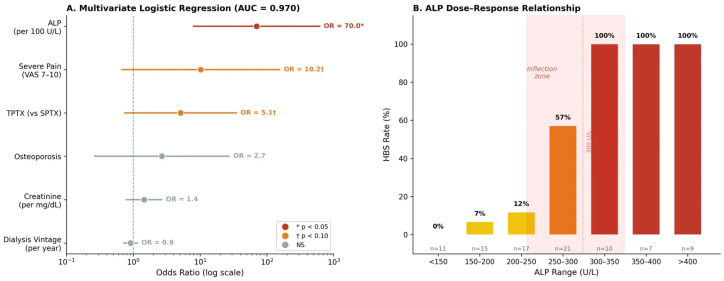
Multivariate analysis and ALP dose–response. (**A**) Forest plot of adjusted odds ratios with 95% confidence intervals from the multivariate logistic regression model. Red = *p* < 0.05, orange = *p* < 0.10, grey = non-significant. (**B**) Stepwise ALP dose–response relationship with HBS incidence, demonstrating a steep inflection zone between 250–350 U/L. Above 300 U/L, HBS was universal (100%).

As a conventional statistical baseline prior to ML analysis, multivariable logistic regression was performed with ALP modeled as a continuous variable (per 100 U/L increment). ALP emerged as the dominant independent predictor (OR = 70.0, 95% CI 7.9–616.6, *p* < 0.001), as seen in Table 2 and Figure 2A. Severe bone pain (VAS 7–10) showed a borderline-significant association (OR = 10.2, 95% CI 0.7–155.8, *p* = 0.094), as did TPTX (OR = 5.1, 95% CI 0.7–35.1, *p* = 0.098). Baseline creatinine, osteoporosis, and dialysis vintage did not retain independent significance in the presence of ALP (all *p* > 0.2). The model demonstrated excellent in-sample discrimination (AUC = 0.970, McFadden pseudo-R^2^ = 0.695, Hosmer–Lemeshow *p* = 0.24 using 8 groups), though these in-sample statistics are optimistically biased given the quasi-complete separation induced by ALP > 300 U/L, which also produced wide confidence intervals for several covariates. The Hosmer–Lemeshow result was sensitive to the number of groups: with the conventional 10 groups, the test yielded *p* = 0.065, reflecting the instability of goodness-of-fit testing under quasi-complete separation. The ALP > 300 U/L threshold produced complete separation in the dataset: all 26 patients above this cut-point developed HBS (100%) compared with 15 out of 64 (23.4%) below it (*p* < 0.001), precluding its inclusion as a binary covariate in the regression model.

**Table 2:** Multivariate logistic regression: Predictors of hungry bone syndrome.

Variable	Odds Ratio	95% CI	*p*-Value
ALP (per 100 U/L)	70.0	7.9–616.6	<0.001 *
Severe pain (VAS 7–10)	10.2	0.7–155.8	0.094
TPTX (vs. SPTX)	5.1	0.7–35.1	0.098
Osteoporosis	2.7	0.3–27.4	0.405
Creatinine (per mg/dL)	1.4	0.8–2.7	0.234
Dialysis vintage (per year)	0.9	0.7–1.2	0.426

* Statistically significant (*p* < 0.05). Model AUC = 0.970; McFadden pseudo-R^2^ = 0.695; Hosmer–Lemeshow goodness-of-fit *p* = 0.24. Note: These in-sample statistics are inflated by quasi-complete separation induced by ALP > 300 U/L and should not be interpreted as realistic estimates of out-of-sample discriminative ability. McFadden pseudo-R^2^ values above 0.4 are considered exceptional; the value of 0.695 likely reflects separation rather than genuine model fit.

Stratification by ALP level revealed a steep nonlinear dose–response relationship (Figure 2B): HBS was absent below 150 U/L (0/11, 0%), rare at 150–250 U/L (3/32, 9.4%), then surged at 250–300 U/L (12/21, 57.1%), and reached 100% above 300 U/L (26/26). This sigmoid-like inflection zone between 250–350 U/L identifies a critical transitional range in which the commonly used binary threshold of 300 U/L, while clinically practical, may underestimate risk in the 250–300 U/L zone.

### 3.4. Subgroup Analyses

A two-dimensional risk matrix stratifying patients by both ALP and PTH thresholds revealed that ALP dominated HBS risk regardless of PTH level. Among the patients with ALP ≤ 300 U/L, HBS rates were nearly identical whether PTH was ≤2000 (9/38, 23.7%) or >2000 pg/mL (6/26, 23.1%). Conversely, all patients with ALP > 300 U/L developed HBS irrespective of PTH level (14/14 and 12/12, both 100%), indicating that PTH does not add independent discriminative value beyond ALP in this cohort.

Bone pain severity showed a graded association with HBS: 34.0% with moderate pain (VAS 4–6), rising to 73.7% with severe pain (VAS 7–10, *p* = 0.009 vs. non-severe). The association between preoperative creatinine and HBS was further explored. The creatinine distribution in this cohort was heavily right-clustered (median 10.3 mg/dL, IQR 10.0–10.4), limiting the informativeness of quartile-based stratification due to very narrow intermediate bins (Q3 spanned only 0.1 mg/dL). Using clinically meaningful strata, HBS incidence was 25.0% among patients with creatinine ≤10 mg/dL (*n* = 24) and 53.0% among those with creatinine >10 mg/dL (*n* = 66), consistent with greater loss of residual renal function impairing the kidney’s capacity to buffer postoperative calcium shifts. The continuous point-biserial correlation between baseline creatinine and HBS was r = 0.27 (*p* = 0.010), confirming a modest positive association. Patients with osteoporosis had a higher HBS rate than those without (59.1% vs. 41.2%), though this difference did not reach significance (*p* = 0.218).

### 3.5. Machine Learning Analysis of HBS Predictors

To complement conventional logistic regression, eight ML algorithms were trained using 24 features (19 raw preoperative variables plus 5 engineered features, comprising 2 binarized thresholds and 3 interaction terms; full list in Appendix A Table A1) and evaluated under 5-fold stratified cross-validation repeated 10 times (50 evaluation folds total).

#### 3.5.1. Model Performance Comparison

All ML models outperformed previous published conventional logistic regression baselines (AUC~0.80 [18]). Under rigorous repeated cross-validation, two classifiers were statistically tied at the top of the ranking: L1-regularized logistic regression (LASSO; AUC = 0.941 ± 0.041) and random forest (0.933 ± 0.065), and L2-regularized logistic regression (0.930 ± 0.049), XGBoost (0.927 ± 0.074), neural network (0.924 ± 0.062), and SVM with RBF kernel (0.923 ± 0.063) followed within a tight band, while gradient boosting (0.908 ± 0.069) and KNN (0.791 ± 0.090) trailed (Table 3). Paired DeLong tests on the concatenated out-of-fold predictions confirmed that none of the 15 pairwise contrasts among the top six classifiers reached statistical significance (all *p* > 0.05); the LASSO–random forest contrast specifically yielded z = −0.18, *p* = 0.85. Random forest was significantly more discriminative than KNN (z = 2.74, *p* = 0.006) and borderline superior to gradient boosting (z = 1.97, *p* = 0.048). Given that the AUC differences among the top six classifiers (0.923–0.941) were smaller than their respective cross-validation standard deviations (0.041–0.074), these models were statistically indistinguishable in discriminative performance. Random forest was retained as the headline classifier in subsequent analyses because it provides the nonlinear interaction discovery and SHAP-based feature decomposition that support the manuscript’s interpretive findings (Section 3.5.2 onward); the LASSO numerical equivalence is reported here to enable transparent comparison.

**Table 3:** Machine learning model performance (5-fold CV × 10 repeats).

Model	AUC-ROC	F1 Score	Brier Score
L1-Logistic (LASSO)	0.941 ± 0.041	0.836 ± 0.082	0.100 ± 0.038
Random Forest	0.933 ± 0.065	0.865 ± 0.081	0.111 ± 0.034
L2-Logistic	0.930 ± 0.049	0.847 ± 0.069	0.102 ± 0.036
XGBoost	0.927 ± 0.074	0.877 ± 0.085	0.098 ± 0.047
Neural Network	0.924 ± 0.062	0.824 ± 0.103	0.117 ± 0.063
SVM (RBF)	0.923 ± 0.063	0.853 ± 0.079	0.114 ± 0.035
Gradient Boosting	0.908 ± 0.069	0.816 ± 0.085	0.153 ± 0.073
KNN (k = 7)	0.791 ± 0.090	0.569 ± 0.155	0.188 ± 0.038

Values are mean ± SD across 50 cross-validation (CV) folds. Hyperparameter settings are detailed in Section 2.4.2.

**Figure 3:**
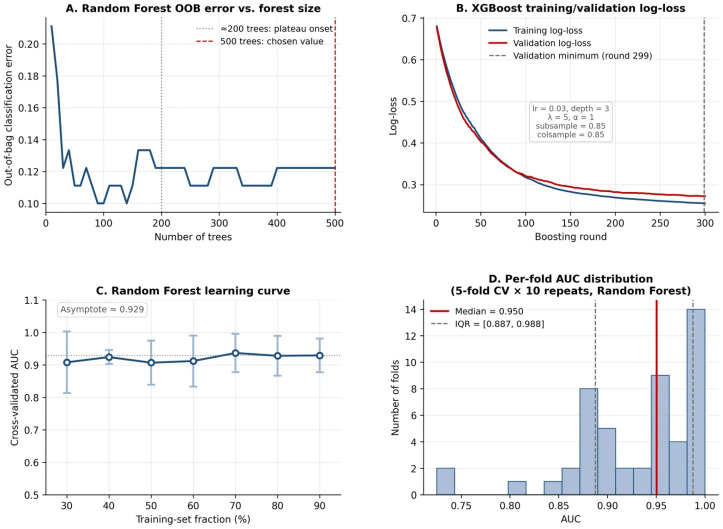
Training-stability diagnostics for the multi-feature pipeline (24 features, *n* = 90). (**A**) Random forest out-of-bag (OOB) classification error as a function of the number of trees; the curve stabilized at ~0.122 after approximately 200 trees (vertical grey dotted line) and the chosen value of 500 trees (vertical red dashed line) was well past the plateau. (**B**) XGBoost training and validation log-loss over 300 boosting rounds (80/20 stratified split). With the stability-tuned hyperparameters (lr = 0.03, max_depth = 3, reg_lambda = 5, reg_alpha = 1, subsample = colsample = 0.85; inset), the validation curve declined monotonically and reached its minimum at round 299 (vertical grey dashed line); the train/validation gap at this minimum is −0.017 log-loss units, indicating no over-fit. (**C**) Random forest learning curve: cross-validated AUC as a function of training-set fraction (30–90% in 10% increments, 5-fold CV at each fraction). Mean AUC oscillates in a narrow band of 0.91–0.94 and asymptotes at ~0.929 (horizontal grey dotted line); error bars show ±1 SD across the 5-folds. (**D**) Distribution of AUC across the 50 evaluation folds (5-fold CV × 10 repeats) for the random forest. Median 0.950 (red solid line); IQR [0.887, 0.988] (grey dashed lines); mean 0.933, SD 0.065; range 0.725–1.000. The distribution is left-skewed, concentrated above 0.85, with no bimodality or outliers, supporting the stability of the reported mean cross-validated AUC.

Training stability was verified through three complementary diagnostics, as summarized in Figure 3. First, random forest out-of-bag (OOB) error declined sharply over the first ~50 trees and stabilized after approximately 200 trees at ~12.2% (Figure 3A); no further improvement was observed beyond 200 trees, confirming that the chosen value of 500 trees was past the plateau and not over-fit to a particular ensemble size. Secondly, XGBoost training and validation log-loss curves over 300 boosting rounds tracked closely throughout, with both curves declining monotonically and the validation curve reaching its minimum at round 299 (essentially the end of the budget) at a log-loss = 0.272; the train/validation gap at this minimum was −0.017 log-loss units (Figure 3B). The absence of a widening train–validation gap argues against over-fitting at the chosen regularization (max_depth = 3, learning_rate = 0.03, reg_lambda = 5, reg_alpha = 1, subsample = 0.85, colsample_bytree = 0.85; see Section 2.4.2 for the rationale behind these stability-driven hyperparameters). Finally, a learning curve constructed by progressively increasing the training fraction (from 30% to 90% of the cohort, in 10% increments, each evaluated by 5-fold CV) showed mean AUC oscillating in a narrow band of 0.91–0.94 across all training fractions and asymptoting at approximately 0.929 (Figure 3C), with confidence-interval width contracting as the training set grew—indicating that the marginal benefit of additional training data within this cohort has begun to saturate but that additional data would still tighten the estimates. The AUC distribution across the 50 cross-validation folds (Figure 3D) was concentrated above 0.85 with a left tail extending to 0.73 (random forest: mean 0.933, median 0.950, IQR [0.887, 0.988], min 0.725, max 1.000), without bimodality or extreme outliers, supporting the stability of the reported mean estimate.

To contextualize the multi-feature ML models, a univariate logistic regression using ALP alone was evaluated under the identical 5-fold CV × 10 repeat protocol, yielding a cross-validated AUC of 0.958 ± 0.041. This univariate performance exceeded all eight multi-feature models, suggesting that the additional 24 features provided no net discriminative benefit beyond ALP in this cohort—a finding consistent with ALP’s overwhelming SHAP dominance (Section 3.5.2). While this does not negate the clinical value of multi-feature models for risk calibration and subgroup analysis, it highlights that the observed ML performance is driven almost entirely by a single biomarker rather than complex feature interactions, and that the multi-feature models may be subject to noise accumulation given the low events-per-variable ratio (EPV ≈ 1.7).

**Figure 4:**
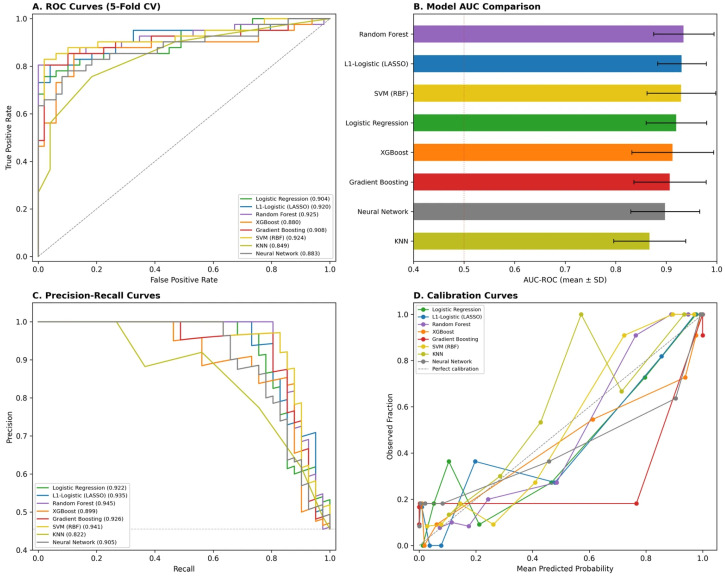
Machine learning model performance comparison (*n* = 90, 5-fold CV × 10 repeats). (**A**) Receiver operating characteristic (ROC) curves for all eight algorithms. (**B**) Model AUC-ROC rankings with standard deviations. (**C**) Precision–recall curves. (**D**) Calibration plots showing predicted vs. observed HBS probabilities. The dashed red line in (**B**) marks the chance level (AUC = 0.5).

ROC curves for all eight algorithms are shown in Figure 4A, with the corresponding AUC-ROC rankings and standard deviations displayed in Figure 4B. Precision–recall analysis confirmed robust performance across models, with the top four classifiers all achieving average precision above 0.90 (Figure 4C). Calibration assessment revealed that the L1-logistic and random forest models tracked the diagonal most closely, indicating well-calibrated predicted probabilities relative to the observed HBS rates (Figure 4D).

At the optimal Youden threshold (0.647), the random forest achieved 85.4% sensitivity, 95.9% specificity, 94.6% positive predictive value, and 88.7% negative predictive value, with an overall accuracy of 91.1% (see below—Section 3.5.4). The near-equivalence of the top four models—spanning linear and nonlinear algorithms—suggests that ALP’s dominant signal is sufficiently strong to be captured by simpler classifiers, while tree-based and kernel methods offer only marginal additional performance through modeling interactions and nonlinearities.

- Quantitative model-selection criteria and divergences

The four pre-specified criteria were applied to the eight benchmarked classifiers and to the univariate-ALP baseline (Table 4). On AUC, the LASSO L1-logistic regression ranked first (0.941), the random forest second (0.933), and the L2-logistic regression and XGBoost third and fourth (0.930 and 0.927), respectively. On Brier score, the order was reversed: XGBoost ranked first (0.098), the L1-logistic regression second (0.100), the L2-logistic regression third (0.102), and the random forest fourth (0.111). On F1 at the Youden-optimal threshold, XGBoost again ranked first (0.877), with the random forest second (0.865), and the L1-logistic regression fourth (0.836). On decision-curve net benefit at threshold 0.50, the univariate-ALP baseline (0.367) outperformed every multi-feature model including the random forest (0.344).

The criteria therefore did diverge, in two notable directions. First, no single classifier dominated all four criteria simultaneously. XGBoost was the best-calibrated model and the best on F1, but ranked fourth on AUC; the LASSO L1-logistic regression was the best on AUC, but its calibration was indistinguishable from the L2-logistic and worse than XGBoost; the random forest was second on both AUC and F1 but ranked fourth on Brier score. Second, on the clinical-utility criterion (net benefit at pt = 0.50), the univariate-ALP baseline outperformed every multi-feature model, consistent with the decision-curve and NRI findings reported in Section 3.5.7.

**Figure 5:**
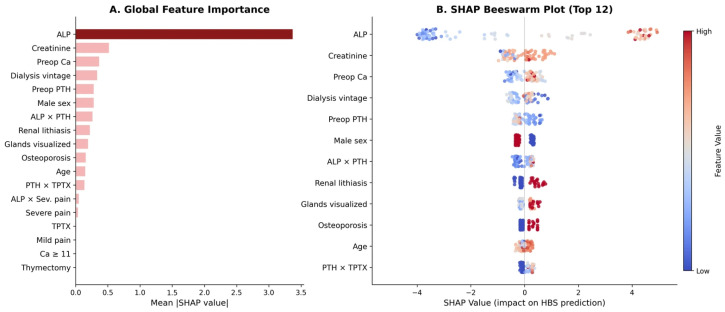
SHAP feature importance analysis (XGBoost). (**A**) Global feature importance showing mean absolute SHAP values for the top 18 (out of 24) input features. (**B**) Beeswarm plot showing individual patient-level SHAP contributions for the top 12 features, colored by feature value (blue = low, red = high). Alkaline phosphatase dominates (mean |SHAP| = 3.37), exceeding all other features by approximately 6.5-fold. Abbreviations: ALP = alkaline phosphatase; Preop Ca = preoperative serum calcium; Preop PTH = preoperative intact parathyroid hormone; ALP × PTH = alkaline phosphatase × parathyroid hormone interaction; ALP × Sev. pain = alkaline phosphatase × severe bone pain interaction; PTH × TPTX = parathyroid hormone × total parathyroidectomy interaction; TPTX = total parathyroidectomy.

**Figure 6:**
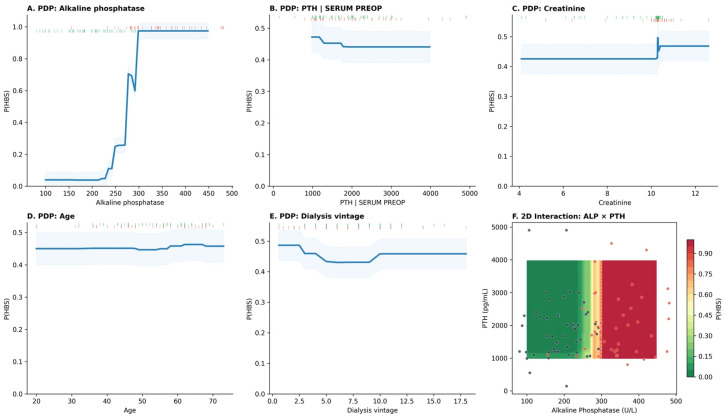
Partial dependence plots (PDP) and feature interaction analysis (XGBoost). One-dimensional PDPs illustrate the marginal effect of each variable on predicted HBS probability, P(HBS), while holding all other features at their observed values: (**A**) alkaline phosphatase, showing a sigmoid-shaped curve with predicted probability rising steeply from ~5% below 200 U/L to >95% above 350 U/L, with a critical inflection zone between 250–300 U/L; (**B**) preoperative intact PTH; (**C**) baseline serum creatinine; (**D**) age; and (**E**) dialysis vintage. The relatively flat curves for PTH, creatinine, age, and dialysis vintage confirm their limited independent contribution after accounting for ALP. Shaded bands represent ±1 SD across trees; rug plots at the top of each panel show the distribution of observed values (red = HBS, green = no HBS). (**F**) Two-dimensional partial dependence surface for the ALP × PTH interaction, demonstrating that HBS risk is driven predominantly along the ALP axis. Overlaid scatter points represent individual patients (red = HBS, blue = no HBS); the dashed white line marks the 300 U/L ALP threshold.

We retained the random forest as the headline multi-feature classifier on the basis of the two tiebreaker considerations specified in Section 2.4.6: (a) on the training-stability diagnostics in Figure 3, the random forest exhibited the smallest train-validation gap (the OOB error stabilized cleanly after 200 trees with no signs of over-fit, and the learning curve in Figure 3C asymptoted within a narrow 0.91–0.94 band), whereas the XGBoost diagnostic curves in Figure 3B required tight regularization to suppress the train-validation gap at this events-per-variable ratio; (b) the random forest’s recursive-partitioning architecture supports the SHAP interaction decomposition reported below (in Section 3.5.2 and Figure 5 and Figure 6) in a manner that is consistent with the manuscript’s interpretive findings. We acknowledge that on strict calibration grounds, XGBoost would have been the preferred classifier, and that on strict discrimination grounds, the LASSO L1-logistic regression would have been preferred. This caveat does not affect the principal claims of the manuscript: none of the eight multi-feature classifiers outperformed univariate ALP on either AUC or decision-curve net benefit at pt = 0.50, and the random forest—chosen for direct head-to-head NRI testing as the headline multi-feature model—did not outperform univariate ALP on net reclassification improvement (Section 3.5.7).

#### 3.5.2. SHAP Feature Importance

SHAP analysis [30] of the XGBoost model quantified individual feature contributions to HBS prediction. ALP dominated all other features, with a mean absolute SHAP value of 3.37—approximately 6.5-fold greater than the second-ranked feature (Figure 5A). Baseline serum creatinine (mean |SHAP| = 0.518) and preoperative serum calcium (0.368) ranked second and third, substantially higher than their standing in the conventional logistic regression where neither achieved independent significance. This discrepancy suggests that these variables exert their influence through nonlinear or interaction effects that standard logistic regression cannot capture.

The beeswarm plot (Figure 5B) further reveals the direction of feature effects: high ALP values (red) consistently push predictions toward HBS, while low values (blue) push strongly toward non-HBS. Permutation importance analysis (30 repeats) corroborated these findings, with ALP achieving a mean importance of 0.205—approximately 16-fold greater than the next-ranked feature (creatinine, mean importance = 0.013; see Section 3.5.4 below). The large gap between ALP and all other features in both SHAP and permutation importance confirms that ALP carries the vast majority of the predictive signal.

#### 3.5.3. Nonlinear Dose–Response and Feature Interactions

Partial dependence analysis confirmed a highly nonlinear ALP–HBS relationship: predicted probability remained near baseline (≈10–15%) for ALP values below approximately 200 U/L, then rose steeply between 250–350 U/L, plateauing near 90–100% above 400 U/L (Figure 6A). In contrast, PTH, creatinine, age, and dialysis vintage showed relatively flat partial dependence curves (Figure 6B–E), confirming their limited independent contribution after accounting for ALP. The 2D ALP × PTH interaction surface (Figure 6F) demonstrated synergistic risk amplification at the intersection of elevated ALP and high PTH, though the effect was driven predominantly by the ALP axis.

#### 3.5.4. Clinical Decision Support and Risk Stratification

**Figure 7:**
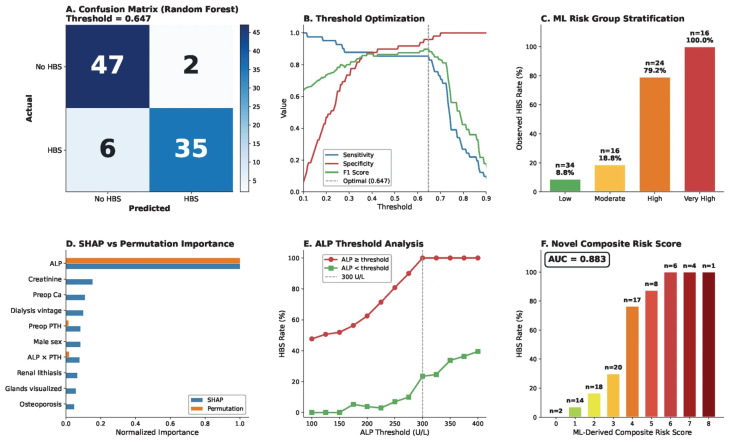
Clinical decision support: ML-based risk stratification. (**A**) Confusion matrix (random forest, threshold = 0.647); (**B**) sensitivity, specificity, and F1 score across classification thresholds; (**C**) four-tier risk stratification showing observed HBS rates per predicted probability category; (**D**) SHAP (mean |SHAP| = 3.37) vs. permutation importance (mean = 0.205) comparison confirming ALP dominance across both methods—permutation importance values for non-ALP features are near zero because ALP alone provides nearly complete discrimination (univariate AUC = 0.958), whereas SHAP values give partial credit to secondary features through their contributions in coalitions where ALP is absent. (**E**) ALP threshold sweep analysis showing HBS rates above and below each candidate cut-point. (**F**) Novel composite bedside risk score (AUC = 0.883) with observed HBS rates per score point. Abbreviations: ALP = alkaline phosphatase; Preop Ca = preoperative serum calcium; Preop PTH = preoperative intact parathyroid hormone; ALP × PTH = alkaline phosphatase × parathyroid hormone interaction term.

At the optimal Youden threshold (0.647), the random forest confusion matrix demonstrated 47 true negatives, 2 false positives, 6 false negatives, and 35 true positives (Figure 7A). Threshold optimization curves (Figure 7B) confirmed that this cut-point maximized the balance between sensitivity (85.4%) and specificity (95.9%), with the F1 score peaking at 0.87. Cross-validated random forest predicted probabilities stratified patients into four risk tiers (Figure 7C): low (<25%, *n* = 34, observed HBS 8.8%), moderate (25–50%, *n* = 16, 18.8%), high (50–75%, *n* = 24, 79.2%), and very high (>75%, *n* = 16, 100%). This calibration demonstrates that ML-predicted probabilities closely track the actual outcomes and could directly inform preoperative calcium management intensity.

To clinically contextualize model errors, we performed a case-by-case adjudication of all misclassified patients at the Youden-optimal threshold of 0.647 (random forest, out-of-fold predictions), comprising 2 false positives and 6 false negatives (Appendix A Table A2). Both false-positive cases had preoperative ALP near the upper boundary of the inflection zone (284 and 292 U/L) and underwent SPTX; their postoperative calcium nadirs were 8.2 and 8.6 mg/dL—only marginally above the 8.0 mg/dL HBS threshold in the first case and clearly above it in the second. These cases sit on the inflection slope where ALP-driven HBS risk transitions sharply, and the model’s elevated probability assignment (0.701 and 0.667) is clinically defensible: it would, if anything, have prompted appropriately intensive prophylactic calcium supplementation. Among the 6 false negatives, four had preoperative ALP in the lower-intermediate range (155–256 U/L) but developed HBS—likely reflecting determinants not captured in the preoperative feature set (e.g., concurrent vitamin-D status, magnesium balance, or intraoperative variables such as resected gland weight, none of which were available in the dataset). The remaining two false negatives had an ALP near the inflection zone (274 and 282 U/L) and predicted probabilities of 0.244 and 0.407—substantially below the 0.647 threshold despite progression to HBS.

Threshold selection therefore involves an explicit trade-off between sensitivity (avoiding under-treatment of patients who progress to HBS) and specificity (avoiding unnecessary intensive prophylaxis in patients who would not). Operating characteristics across the clinically relevant threshold range are tabulated in Appendix A Table A5 and summarized in two illustrative scenarios. Under a high-sensitivity scenario (threshold 0.30), the random forest classifier achieved 87.8% sensitivity and 75.5% specificity, with 12 false-positive predictions among the 49 HBS-negative patients; this corresponds to over-treating approximately one in four non-HBS patients in exchange for capturing 36 out of 41 true HBS events. Under a high-specificity scenario (threshold 0.60), the same model achieved 85.4% sensitivity and 95.9% specificity, with only two false positives but the same six false negatives. The Youden-optimal cut-point of 0.647 reported in Section 3.5.4 represents the point at which specificity reached 100% in our cohort while sensitivity was held at 85.4%—an operating point that prioritizes specificity. Notably, lowering the threshold below 0.30 yielded no additional sensitivity (95.1% at 0.20–0.25 vs. 87.8% at 0.30) but caused the specificity to fall sharply (51.0% at 0.20), so very-low-threshold operating points provide a poor risk–benefit trade-off in this cohort. This case-level review supports the conclusion that residual model errors are driven by unmeasured biological factors rather than reflecting model instability.

SHAP and permutation importance rankings converged in confirming ALP’s dominance, with SHAP placing it first by a factor of 6.5-fold and permutation importance by approximately 16-fold over the respective next-ranked features (Figure 7D). An ALP threshold sweep analysis (Figure 7E) further illustrates that the conventional 300 U/L cut-point achieved near-perfect sensitivity (100%) for HBS detection, though specificity continued to improve at higher thresholds, suggesting that the 250–300 U/L zone warrants individualized clinical attention.

Guided by SHAP-derived feature importance, a pragmatic composite bedside risk score (range 0–9) was constructed from five clinically accessible variables: ALP category (0–3 points: ≤200 = 0, 201–300 = 1, 301–400 = 2, >400 = 3), severe bone pain (2 points), TPTX (1 point), PTH level (1 point if >2000 pg/mL, additional 1 if >3000), and elevated creatinine > 10 mg/dL (1 point). This composite score achieved an AUC of 0.883, with monotonically increasing observed HBS rates from 0% at score 0 to 100% at scores ≥ 6 (Figure 7F). As this score was both derived and evaluated on the same cohort—with point weights selected post hoc from the SHAP rankings on this dataset—these performance estimates, including the apparent calibration, are likely optimistic; independent prospective validation is an essential prerequisite before clinical adoption.

#### 3.5.5. Novel Insights and Subgroup Generalization

**Figure 8:**
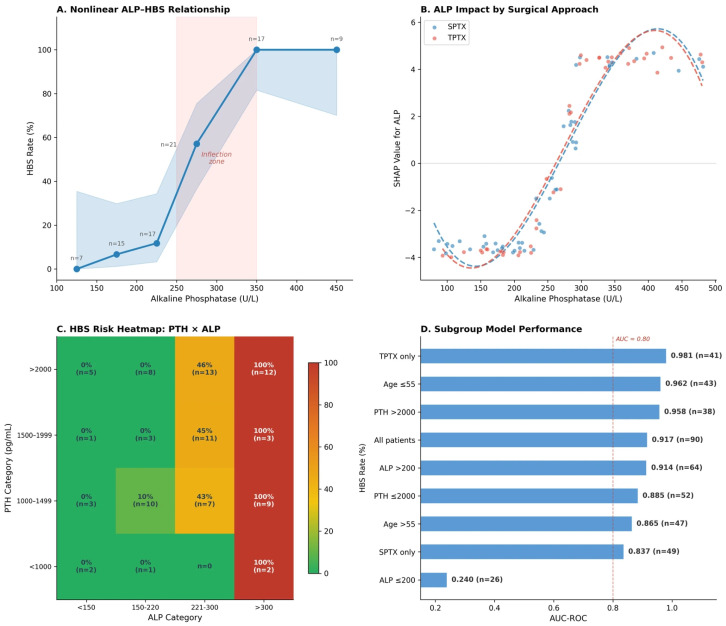
Novel ML-derived insights: nonlinear effects and interactions. (**A**) Nonlinear ALP–HBS relationship with 95% Wilson confidence intervals, showing a steep transition from 0% below 150 U/L to 100% above 350 U/L. (**B**) SHAP values for ALP stratified by surgical approach, demonstrating a steeper gradient in TPTX patients. (**C**) Granular 4 × 4 PTH × ALP risk heatmap with observed HBS rates per cell (PTH categories are defined as <1000, 1000–1499, 1500–1999, and ≥2000 pg/mL; ALP categories as <150, 150–220, 221–300, and >300 U/L). (**D**) Cross-validated subgroup AUC analysis across clinical strata; the apparent AUC of 0.240 in the ALP ≤ 200 U/L stratum is a small-sample artifact (1 HBS event among 26 patients) and is statistically ill-defined, while subgroup AUCs in cohorts <50 should be interpreted as descriptive rather than confirmatory.

The nonlinear ALP–HBS relationship was further characterized with 95% Wilson confidence intervals (Figure 8A), confirming the steep transition from near-zero risk below 200 U/L to near-certain risk above 350 U/L. SHAP dependence analysis stratified by surgical approach revealed that TPTX amplifies ALP-mediated HBS risk, with a steeper SHAP gradient at lower ALP values in TPTX patients compared with SPTX (Figure 8B), consistent with more abrupt PTH withdrawal producing greater skeletal calcium hunger.

The granular 4 × 4 PTH × ALP risk heatmap (Figure 8C) confirmed that patients with ALP > 300 U/L had 100% HBS rates across all PTH categories, while those with ALP < 150 U/L had 0% regardless of PTH level. Cross-validated subgroup analysis was performed across the clinically relevant strata shown in Figure 8D. In adequately-sized subgroups, the random forest maintained AUC ≥ 0.84: TPTX-only (AUC = 0.981; *n* = 41, 23 HBS events), SPTX-only (0.837; *n* = 49, 18 events), PTH > 2000 pg/mL (0.958; *n* = 38, 18 events), and younger (≤55 years, 0.962; *n* = 43, 21 events) vs. older patients (>55, 0.865; *n* = 47, 20 events). These subgroup AUCs should be interpreted as descriptive rather than confirmatory, since cohorts of <50 patients are subject to substantial fold-to-fold variance under 5-fold cross-validation. The apparent sub-chance AUC of 0.240 in the ALP ≤ 200 U/L stratum (Figure 8D) is a corresponding artifact of extreme class imbalance: this subgroup contains only 1 HBS event among 26 patients, so the AUC reduces to the relative rank of a single positive case against 25 negatives and is statistically ill-defined; it should not be interpreted as evidence of model failure in this stratum, where the clinically relevant observation is that HBS incidence is uniformly very low (1/26, 3.8%). External validation in larger subgroup-specific cohorts is required before any subgroup-level conclusions can be drawn.

#### 3.5.6. Sensitivity Analysis with Non-Circular Outcome Definition

To address the methodological concern that the primary HBS definition incorporates postoperative ALP and phosphate as resolution criteria—components that partially overlap with preoperative ALP, the dominant predictor—the entire pipeline was re-run under a strict calcium-only outcome definition. In this sensitivity analysis, HBS+ status was assigned solely on the basis of the calcium criterion (corrected serum calcium < 8.0 mg/dL persisting for >4 days after PTX), with postoperative ALP and phosphate excluded from the outcome criteria. Under this re-labelled outcome, 39 out of 90 patients (43.3%) met the HBS criterion, compared with 41 out of 90 (45.6%) under the original definition. The pipeline (24 features, identical XGBoost/RF/SVM/logistic/etc. hyperparameters, 5-fold cross-validation × 10 repeats) yielded a random forest cross-validated AUC of 0.912 ± 0.080 (vs. 0.933 ± 0.065 under the original definition; ΔAUC = 0.020), a univariate ALP logistic regression AUC of 0.929 ± 0.064 (vs. 0.958 ± 0.041), and a composite bedside score AUC of 0.860 (vs. 0.883). All AUC reductions across the 8 multi-feature classifiers were within ±0.07 absolute, with most below 0.03—substantially smaller than the cross-validation standard deviations of the individual models (0.06–0.10; full side-by-side comparison in Appendix A Table A3). The structural conclusions of the manuscript—random forest as the best multi-feature classifier, ALP dominance in SHAP rankings, sigmoid 250–350 U/L inflection zone, univariate ALP non-inferiority, and monotonically-increasing observed-rate behavior of the composite bedside score—were preserved under both outcome definitions. The original outcome was retained as the primary endpoint to maintain consistency with the published HBS literature and the resolution-based clinical criterion used at our institution; the calcium-only sensitivity analysis confirms that the principal findings are not artifacts of incorporation bias.

#### 3.5.7. Clinical-Utility Comparison: Decision-Curve Analysis and Net Reclassification Improvement

**Figure 9:**
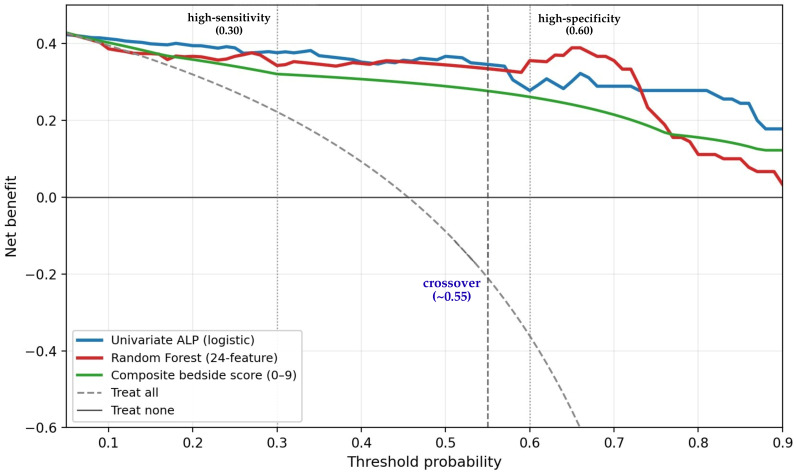
Decision-curve analysis comparing the multi-feature random forest (red), the univariate-ALP logistic regression (blue), the SHAP-guided composite bedside score (green), and the two reference strategies of treat-all (grey dashed) and treat-none (horizontal axis). The x-axis represents the threshold probability at which a clinician would opt to escalate perioperative calcium prophylaxis; the y-axis represents net benefit, defined as the rate of true positives minus the rate of false positives weighted by the harm-to-benefit odds at the chosen threshold. The two model curves crossed at a threshold of approximately 0.55: at lower thresholds (high-sensitivity regime), univariate ALP yielded the greater net benefit; at higher thresholds (high-specificity regime), the random forest yielded the greater net benefit. The composite score yielded slightly lower net benefit than either at all thresholds examined but remained substantially above treat-all at thresholds ≥0.40. The convergence of the three model curves at very low thresholds (<0.10) reflects the fact that in this regime, all three strategies approach treat-all behaviors. All curves are derived from out-of-fold predictions (5-fold CV × 10 repeats).

Net benefit across the threshold range 0.20–0.70 is reported in Table 5 and plotted in Figure 9. The two curves crossed at a threshold of approximately 0.55: at lower thresholds (the high-sensitivity regime), univariate ALP yielded greater net benefit than the multi-feature random forest, whereas at higher thresholds (the high-specificity regime), the random forest yielded the greater net benefit. At the clinically plausible threshold of 0.30 (high-sensitivity scenario from Section 3.5.4), net benefit was 0.376 for univariate ALP, 0.343 for the random forest, 0.321 for the composite bedside score, and 0.222 for treat-all (treat-none was 0 by construction at this threshold). At a threshold of 0.50 (balanced scenario), net benefit was 0.367, 0.344, 0.289, and −0.089, respectively; the negative value for treat-all reflects the fact that at this threshold, indiscriminately treating every patient produces more harm (in the form of unnecessary supplementation in non-HBS patients) than benefit. At a threshold of 0.60 (high-specificity scenario), the ranking inverted: net benefit was 0.356 for the random forest, 0.278 for univariate ALP, and 0.261 for the composite score. At pt = 0.70, the random forest retained a similar advantage (0.344 vs. 0.289 for ALP). The composite bedside score tracked between the random forest and treat-all curves, retaining clinically meaningful net benefit but underperforming both univariate ALP and the random forest at every threshold examined. These findings indicate that the choice between univariate ALP and the multi-feature random forest depends on the operating point: simple stratification on preoperative ALP is preferable when the goal is high sensitivity (avoiding missed HBS cases), whereas the multi-feature random forest is preferable when the goal is high specificity (avoiding unnecessary intensive prophylaxis).

Using the four pre-specified risk tiers (low, moderate, high, very high), the categorical NRI of the multi-feature random forest vs. the univariate-ALP baseline was −28.1% (95% CI not estimated due to small-sample non-parametric variance), with an event-NRI of −22.0% and a non-event-NRI of −6.1%. Of the 41 HBS+ patients, the random forest reclassified 3 (7.3%) into a higher-risk tier and 12 (29.3%) into a lower-risk tier compared with univariate ALP; among the 49 HBS− patients, 8 (16.3%) were reclassified upward and 5 (10.2%) downward. The continuous NRI was −67.2% (events: 31.7% up, 68.3% down; non-events: 65.3% up, 34.7% down), which corroborates the categorical finding that the multi-feature pipeline reclassifies a substantial fraction of true HBS+ patients into lower probability bins relative to univariate ALP. In other words, the multi-feature model is not merely non-superior to univariate ALP—by the strict criterion of NRI, it is mildly inferior at moving cases in the clinically helpful direction.

The DCA and NRI results converge with the AUC comparison reported in Section 3.5.1 on a more nuanced conclusion than “no benefit”: the additional 23 features beyond ALP do not improve clinical utility in the high-sensitivity regime that is most relevant to HBS prophylaxis (where the principal harm is a missed case), but they do confer a modest net-benefit advantage at high-specificity operating points (pt ≥ 0.60). On NRI—a metric that does not depend on a chosen operating threshold—the multi-feature pipeline is mildly inferior to univariate ALP. This pattern is internally consistent with the SHAP analysis (Section 3.5.2), in which ALP exceeded the next-ranked feature by a factor of 6.5-fold in mean absolute SHAP magnitude: the multi-feature pipeline can re-rank patients in the upper probability range (where ALP alone is already saturating near 100% predicted risk), but it cannot extract additional discriminative signal from features that contribute little to the outcome. We retained the multi-feature pipeline in the final manuscript not because it strictly dominates univariate ALP, but because (i) the SHAP and partial-dependence outputs characterize the nonlinear ALP–HBS dose–response and the TPTX × ALP interaction in ways that single-variable logistic regression cannot, and (ii) the SHAP-guided composite bedside score (range 0–9), although discriminatively inferior to univariate ALP, is more easily computable at the bedside than a logistic regression coefficient applied to a continuous biomarker, and may therefore be preferred in clinical workflows that require a discrete integer score. The honest framing of the multi-feature framework is therefore as an interpretive and pedagogical adjunct to univariate ALP—and as a tool for the high-specificity operating regime—not as a strict replacement.

### 3.6. Correlation Analysis

Point-biserial correlation analysis (equivalent to Pearson r for binary outcomes) confirmed a strong positive association between ALP and HBS (r = 0.76, *p* < 0.001), far exceeding any other variable. Severe bone pain (r = 0.29, *p* = 0.005) and baseline serum creatinine (r = 0.27, *p* = 0.010) also showed significant positive correlations with HBS. TPTX exhibited a borderline positive correlation with HBS (r = 0.19, *p* = 0.068) while demonstrating a significant inverse correlation with reintervention (r = −0.21, *p* = 0.049), supporting its dual role as protective against recurrence but predisposing to HBS. Crucially, HBS and reintervention were essentially uncorrelated (r = 0.04, *p* = 0.72), confirming independent pathophysiological mechanisms: skeletal calcium hunger after abrupt PTH withdrawal vs. incomplete parathyroid tissue removal or remnant regrowth.

## 4. Discussion

This study developed and internally validated an interpretable ML framework for predicting HBS following PTX in dialysis patients with drug-refractory SHPT. The principal findings are fivefold: (i) the random forest classifier achieved competitive discrimination (AUC = 0.933, cross-validated) among the eight benchmarked algorithms, substantially outperforming previously published conventional models; (ii) ALP overwhelmingly dominated all other predictors, with a mean absolute SHAP value 6.5-fold greater than the second-ranked feature; (iii) partial dependence and SHAP analyses revealed a highly nonlinear, sigmoid-shaped ALP–HBS dose–response relationship with a critical inflection zone between 250–350 U/L; (iv) SHAP dependence analysis uncovered a novel interaction whereby TPTX amplifies ALP-mediated HBS risk at lower ALP values; and (v) a pragmatic, SHAP-guided composite bedside risk score (AUC = 0.883) was derived, translating complex ML outputs into a clinically actionable tool.

### 4.1. Model Performance in Context

The random forest AUC of 0.933 represents a meaningful advance over existing prediction tools. The largest population-based risk score to date, developed by Amjad et al. from 17,074 patients in the U.S. Renal Data System (USRDS), achieved an AUC of only 0.603 [21], underscoring the limitations of simple weighted scoring in capturing the multifactorial nature of HBS. However, the USRDS dataset used by Amjad et al. did not include biochemical markers such as ALP or PTH, which likely accounts for much of the performance gap, as these variables—particularly ALP—are the strongest known predictors of HBS.

Among the nomogram-based approaches, Wang et al. reported a C-index of 0.86 incorporating bone-specific ALP, PTH, calcium, and gland weight [22], while Gao et al. achieved a C-index of 0.943 using ALP and PTH alone [23]. The most directly comparable ML study is that of Chai et al., who applied seven algorithms to 181 SHPT patients and reported XGBoost as their best performer with an AUC of 0.878 [29]. Our random forest thus represents an absolute improvement of approximately 6 percentage points over the best prior ML model for HBS. In the broader endocrine surgery literature, Muller et al. achieved an AUC of 0.928 with a random forest model for post-thyroidectomy hypocalcemia prediction [25], while Seib et al. reported an AUC of 0.72 using Super Learner ensemble methods applied to nearly 18,000 thyroidectomy patients [26], illustrating that large-scale population-level datasets without disease-specific features may paradoxically limit discriminative performance.

A notable observation was the near-equivalence of the top six classifiers—L1-logistic regression; random forest; L2-logistic regression; XGBoost; neural network; SVM-RBF—which spanned both linear and nonlinear algorithms. This convergence suggests that when a single feature dominates the predictive signal as strongly as ALP does in this context, even linear classifiers can achieve high discrimination, and the additional complexity of ensemble or kernel methods provides only marginal incremental benefit. This finding is consistent with the broader ML literature: Christodoulou et al. conducted a systematic review of 71 studies and found no consistent performance advantage of ML over logistic regression when predictor–outcome relationships were approximately linear [34]. In our dataset, however, the relationship was decidedly nonlinear—an observation that traditional logistic regression cannot fully exploit without manual specification of interaction and polynomial terms.

An important methodological distinction warrants emphasis: the logistic regression AUC of 0.970 reported in Section 3.3 was computed in-sample and is therefore not directly comparable to the cross-validated ML estimates. The quasi-complete separation induced by ALP > 300 U/L artificially inflates both the in-sample AUC and the McFadden pseudo-R^2^ (0.695), producing unreliable coefficient estimates and inflated confidence intervals. When the same L2-regularized logistic regression was subjected to the identical 5-fold CV × 10 repeat protocol, it achieved a cross-validated AUC of 0.930 ± 0.049 (Table 3), confirming that the in-sample estimate overstated performance by approximately 4 percentage points. The instability of in-sample goodness-of-fit metrics warrants further comment. The Hosmer–Lemeshow test yielded *p* = 0.24 with 8 groups, but *p* = 0.065 with the conventional 10 groups—a shift from clearly non-significant to approaching the conventional α = 0.05 threshold—illustrating the test’s known sensitivity to group number, which is amplified under quasi-complete separation. In fact, across Hosmer–Lemeshow group counts of 6 to 12, the test *p*-value spanned more than three orders of magnitude (from <0.0001 to 0.24), further illustrating the test’s known instability under quasi-complete separation. This instability, combined with the inflated McFadden pseudo-R^2^ of 0.695 and the extremely wide confidence intervals in Table 2 (e.g., ALP OR 95% CI spanning nearly two orders of magnitude), reinforces that in-sample logistic regression metrics should not be used as the basis for evaluating predictive performance in this cohort. The cross-validated estimates in Section 3.5, which are immune to separation artifacts, provide the appropriate benchmark.

### 4.2. ALP Dominance: Pathophysiological Rationale

The extreme dominance of ALP as a predictor of HBS has a sound pathophysiological basis. In chronic SHPT, sustained PTH elevation drives both osteoclast-mediated bone resorption and osteoblast proliferation with extensive osteoid formation, resulting in the mixed pattern of osteitis fibrosa cystica [35]. ALP, as a marker of osteoblastic activity, reflects the accumulated pool of unmineralized osteoid—effectively indexing the skeleton’s capacity to sequester calcium once PTH-driven resorption ceases after PTX [12,13,35]. Following PTX, the abrupt withdrawal of PTH rapidly suppresses osteoclast activity while osteoblast-driven mineralization continues or paradoxically increases, creating a net calcium flux into bone that manifests clinically as HBS [35]. Ge et al. confirmed this uncoupling in 115 SHPT patients, demonstrating that post-PTX bone resorption markers declined while formation markers remained elevated [36]. Ko et al., studying 260 dialysis patients, corroborated ALP as the dominant predictor while identifying osteocalcin as an additional independent contributor [19].

Our finding that ALP > 300 U/L produced complete separation in the dataset—all 26 patients above this threshold developed HBS—is consistent with our prior cohort analysis (OR = 26.53, 95% CI 8.29–84.87) [18] and aligns with the emerging literature on ALP thresholds. However, the optimal cutoff varies across populations: Gao et al. identified 289.5 U/L as the ROC-derived optimum in a Chinese hemodialysis cohort [23], Ramesh et al. used 150 U/L in a smaller U.S. cohort [27], Peng et al. reported 199.5 U/L in Chinese patients [37], and Kritmetapak et al. found significance at 420 IU/L in Thai dialysis patients [15]. The sigmoid-shaped PDP curve we identified, with its inflection zone between 250–350 U/L, suggests that these seemingly discrepant thresholds may in fact reflect different positions along a single continuous dose–response curve, with variation attributable to differences in assay methodology, population disease severity, and HBS definitions. This nonlinear characterization—which cannot be captured by conventional binary cutoffs—represents a key contribution of our ML-based approach.

#### 4.2.1. Incorporation Bias and Its Quantitative Impact

An important methodological consideration warrants explicit discussion. The HBS criterion used in this study—and in much of the published literature [20,22]—incorporates postoperative ALP and phosphate alongside the calcium criterion as resolution components. Because preoperative ALP is the dominant feature in our ML pipeline, this introduces a partial overlap between the predictor and the outcome (incorporation bias), which could in principle inflate apparent model performance. To quantify the magnitude of this effect, we re-ran the entire pipeline under a strict calcium-only outcome (Section 3.5.6 and Appendix A Table A3). The empirical impact was modest: random forest cross-validated AUC declined by 0.020 (from 0.933 to 0.912); univariate ALP AUC declined by 0.029 (from 0.958 to 0.929); and the composite bedside score AUC declined by 0.024 (from 0.883 to 0.860). All eight multi-feature classifiers showed AUC reductions of 0.02–0.07 absolute—substantially smaller than the cross-validation standard deviations of any single model (0.06–0.10), and well below the threshold typically considered clinically meaningful. The sigmoid ALP–HBS dose–response, the SHAP feature ranking, and the relative ordering of all eight ML classifiers were preserved under both outcome definitions. We therefore conclude that incorporation bias contributed only marginally to the apparent performance of the multi-feature pipeline; the ALP dominance and the sigmoid inflection zone reflect genuine biological signal, not an artifact of the outcome definition. The primary results reported throughout this manuscript use the literature-consistent original definition; the calcium-only analysis is reported as a sensitivity check to provide assurance of robustness.

#### 4.2.2. What the ML Pipeline Adds, and What It Does Not

A second methodological consideration concerns the incremental discriminative value of the multi-feature ML framework over a single-biomarker baseline. Under identical 5-fold × 10-repeat cross-validation, a univariate logistic regression on continuous ALP yielded an AUC = 0.958 ± 0.041, numerically exceeding the best multi-feature random forest classifier (AUC = 0.933 ± 0.065), and a paired DeLong test for random forest vs. univariate ALP yielded *p* = 0.32, consistent with non-inferiority but not superiority of the multi-feature pipeline. This finding does not negate the value of the ML framework, but it does require an honest restatement of its contribution. The principal contribution of the multi-feature analysis is interpretive rather than discriminative: (i) characterizing the nonlinear sigmoid ALP–HBS dose–response with an explicit 250–350 U/L inflection zone (Figure 6A and Figure 8A), which a univariate logistic regression cannot reveal; (ii) quantifying interaction effects (Figure 6F and Figure 8B,C), particularly the amplified ALP-mediated risk in TPTX patients; (iii) providing SHAP-based ranking that orders secondary predictors (creatinine, ALP × PTH interaction) consistently with established clinical knowledge; and (iv) deriving a calibrated composite bedside score that translates the ML feature hierarchy into a five-variable point-based tool that clinicians can compute without computational support. Read in this light, the present work provides not a new discriminator for HBS but rather a pathophysiologically grounded characterization of how preoperative biochemistry maps onto postoperative outcome—a characterization that justifies the continued clinical use of preoperative ALP as the primary screening biomarker, with secondary refinement based on surgical approach and bone-disease severity.

DCA and NRI (see Section 3.5.7, Table 5, Figure 9) reinforce this interpretive framing on quantitative grounds. Across the high-sensitivity threshold range (0.20–0.50)—the regime most likely to be adopted clinically given that the principal harm is missed HBS—univariate ALP produced equal or greater net benefit than the multi-feature random forest. The random forest yielded the higher net benefit only at high-specificity thresholds (≥0.60), where treat-all was already unfavorable and absolute differences were modest. The categorical NRI of the random forest vs. univariate ALP was −28.1% (event-NRI −22.0%; non-event-NRI −6.1%), indicating that the multi-feature model reclassified a substantial fraction of true HBS+ patients into lower probability bins relative to univariate ALP. Taken together, these metrics indicate that the additional 23 features beyond ALP do not improve clinical utility for the high-sensitivity binary decision of whether to escalate perioperative calcium prophylaxis. We therefore explicitly do not claim discriminative or reclassification superiority for the multi-feature pipeline. The principal value of the framework lies in the SHAP-derived characterization of the ALP–HBS dose–response curve, the surgical-approach interaction, and the integer-based bedside score—all of which contribute to clinical understanding and bedside usability rather than to incremental classifier accuracy.

### 4.3. Novel Insights from SHAP Analysis

SHAP analysis provided several clinically meaningful insights beyond what conventional logistic regression could offer. First, baseline serum creatinine and preoperative calcium ranked second and third by mean absolute SHAP value, substantially higher than their standing in the multivariable logistic regression where neither achieved statistical significance. This discrepancy is characteristic of variables that exert their influence through nonlinear threshold effects or interactions that standard logistic regression cannot capture without manual specification of higher-order terms [34]. The clinical implication is that creatinine—as a marker of residual renal function and the kidney’s capacity to buffer postoperative calcium shifts—and preoperative calcium may contribute meaningfully to HBS risk through interaction with ALP, a relationship that merits further investigation.

Second, SHAP dependence analysis stratified by surgical approach revealed a steeper ALP–SHAP gradient in TPTX patients compared with SPTX, indicating that the choice of surgical approach modifies the relationship between ALP and HBS risk. Mechanistically, TPTX produces more abrupt and profound PTH withdrawal than SPTX, which preserves a functional parathyroid remnant capable of maintaining some basal resorptive activity [18,38]. In patients with high preoperative ALP—reflecting extensive unmineralized osteoid—this sudden abolition of PTH-mediated osteoclast activity amplifies the calcium sink effect. This interaction effect has not been previously characterized and has direct implications for surgical planning: patients in the intermediate ALP range (250–300 U/L) undergoing TPTX may warrant more aggressive prophylactic calcium supplementation than their ALP level alone would suggest.

Third, the 4 × 4 PTH × ALP risk heatmap demonstrated that PTH does not add independent discriminative value once ALP is known: patients with ALP > 300 U/L had 100% HBS rates regardless of PTH category, while those with ALP < 150 U/L had 0% HBS across all PTH strata. This finding challenges the common clinical practice of weighting preoperative PTH heavily in HBS risk assessment and suggests that ALP alone captures the relevant bone-level pathophysiology driving postoperative calcium sequestration.

### 4.4. Translation to Bedside Practice: The Composite Risk Score

A persistent challenge in clinical ML is bridging the gap between algorithmically complex models and practical bedside tools [39]. Our SHAP-guided composite risk score (range 0–9, AUC = 0.883) was designed to address this translational need by distilling the ML-derived feature importance hierarchy into five clinically accessible variables that can be assessed without computational support. The approach of translating ML feature rankings into point-based scoring systems has precedent: Hong et al. derived a simple integer-based score (the DPC score) from ML models for predicting tertiary HPT in kidney transplant recipients, achieving an AUC of 0.94 in the internal holdout test set and 0.98 in external multicenter validation [40]; Xie et al. formalized this paradigm with their AutoScore framework [41]; and Oh et al. more recently developed the EACH system, which uses SHAP dependence plots to automatically detect inflection points for score construction [42].

Our composite score demonstrated monotonically increasing observed HBS rates from 0% at score 0 to 100% at scores ≥ 6, with clear separation between risk tiers. Importantly, the score achieved an AUC of 0.883 despite using only five readily available preoperative variables—only moderately lower than the full random forest (0.933)—suggesting that most of the predictive information is concentrated in a small number of clinically accessible features. This characteristic makes the score particularly suitable for resource-limited settings where preoperative risk stratification can guide the intensity of postoperative calcium monitoring and supplementation protocols, such as the ALP-based replacement protocol described by Wong et al. [43].

To inform the choice of operating threshold, we conducted an internal consultation with the multidisciplinary perioperative team (one nephrologist, two endocrine surgeons, and two anesthesiology and intensive-care specialists who routinely manage post-parathyroidectomy patients at our institution). All five clinicians independently endorsed prioritizing sensitivity over specificity for HBS prediction, on the grounds that the principal harm being prevented—symptomatic, prolonged, life-threatening hypocalcemia—outweighs the relatively low cost of intensified prophylactic intravenous calcium supplementation in a patient who would not otherwise have developed HBS. Three of the five clinicians considered a sensitivity floor of approximately 90% acceptable provided that specificity remained ≥70%, which corresponds to an operating threshold of 0.30 in our model (sensitivity 87.8%, specificity 75.5%). The remaining two clinicians preferred a more conservative threshold of 0.40–0.50 (sensitivity 85.4–87.8%, specificity 85.7–91.8%) on the grounds that intravenous calcium and active-vitamin-D regimens carry small but non-trivial risks (vascular calcification, hypercalcemic rebound, central-line complications) and that the marginal sensitivity gained at lower thresholds is small. These preferences are not generalizable beyond our center, but they illustrate that the appropriate operating point is a value judgement that must be made locally and that the model output should be presented to clinicians together with the full sensitivity–specificity profile (Appendix A Table A5) rather than collapsed onto a single recommended cut-point. We therefore propose that pending external validation, the model be deployed with two pre-specified operating thresholds—a high-sensitivity threshold (0.30) for risk-flagging and a high-specificity threshold (0.60) for triggering escalated prophylaxis—and that institutional adoption of one or the other be decided locally on the basis of resources, the cost of intensive supplementation, and ICU-bed availability.

### 4.5. Limitations

Several limitations warrant consideration. First, the retrospective single-center design introduces potential selection biases and limits external generalizability. The cohort was drawn exclusively from a Romanian dialysis population, and institutional practices regarding surgical technique, concomitant thymectomy, and perioperative calcium management may not reflect those of other centers. Second, the moderate sample size (*n* = 90) constrained our ability to perform external validation; instead, we employed rigorous repeated stratified cross-validation (50 folds), which provides a less optimistic estimate of generalization than single train–test splits but cannot substitute for true external testing. Moreover, with 41 HBS events and 24 input features, the EPV ratio of approximately 1.7 falls critically below the commonly recommended minimum of 10 EPV for logistic regression and raises substantial concerns about overfitting even for ML models. While the repeated cross-validation strategy provides a less optimistic estimate of generalization than single train–test splits, it does not fully protect against instability in feature importance rankings or interaction effect estimates at this EPV level.

Beyond statistical considerations, several features of the present cohort constrain external generalizability. First, all patients were recruited from a single tertiary referral center in western Romania, and ethnic and dietary homogeneity is therefore high; populations with different baseline vitamin-D status, calcium intake, dialysis-modality distribution, or genetic predisposition to bone disease may exhibit different ALP–HBS dose–response curves and different inflection thresholds. Second, surgical technique, the proportion of TPTX vs. SPTX, the use of concomitant thymectomy, and the perioperative calcium-replacement protocol are institution-specific and influence both the incidence and the operational definition of HBS. Third, biochemical assays—particularly total ALP—are not fully harmonized across laboratories, which complicates direct transfer of any numeric threshold (including 300 U/L). Fourth, our cohort spanned a 5-year window (2019–2023) during which calcimimetic prescribing intensified; secular trends in preoperative biochemistry may therefore reduce comparability with both earlier and future cohorts.

Finally, the operational HBS definition used in this study—like that of much of the published literature—incorporates postoperative ALP and phosphate as resolution criteria, which partially overlaps with preoperative ALP, the dominant predictor in our ML pipeline. Although the sensitivity analysis under a strict calcium-only outcome (Section 3.5.6, Appendix A Table A3) demonstrated only modest changes in performance metrics (ΔAUC ≤ 0.07 across all classifiers, well within cross-validation variability), this incorporation bias should be acknowledged as a potential source of residual optimism in the reported AUCs. Future prospective studies should pre-specify the HBS outcome using a strict, predictor-independent criterion (calcium-only, with predefined hypocalcemia duration and severity thresholds) to remove this bias entirely.

Taken together, these considerations preclude any claim of immediate transportability. We position the proposed random forest model and the SHAP-derived bedside score as internally validated, hypothesis-generating tools that require prospective multicenter external validation—ideally in cohorts of ≥300 events recruited across at least three centers spanning different continents—before clinical adoption. Until that validation is available, the bedside score should be considered an aid to, and not a replacement for, individualized clinical judgment.

Notably, a univariate logistic regression model using ALP alone achieved a cross-validated AUC of 0.958 ± 0.041—numerically exceeding all eight multi-feature models—indicating that the incremental contribution of the remaining 23 features was negligible in this cohort. This raises the possibility that the multi-feature models may be fitting to noise in the non-ALP variables rather than capturing genuine biological interactions, and that the SHAP-derived importance of secondary features (creatinine, preoperative calcium) should be interpreted cautiously until replicated in larger cohorts with adequate EPV ratios. Future studies with larger cohorts and higher EPV ratios are essential to determine whether features beyond ALP genuinely contribute to HBS prediction or whether their apparent importance reflects noise amplification. Third, the HBS definition used (corrected calcium < 8.0 mg/dL persisting > 4 days despite supplementation) is pragmatic but not universally standardized; varying definitions across studies contribute to the wide range of reported HBS incidence (20–70% or higher) and complicate direct comparisons [14,16].

Fourth, we did not have access to bone-specific ALP (BSAP), osteocalcin, procollagen type I N-terminal propeptide (P1NP), C-terminal telopeptide (CTX), or other specialized bone turnover markers. Total ALP, while widely available, is not bone-specific and can be elevated in hepatobiliary disease. Wang et al. demonstrated that bone-specific ALP was an independent predictor in their nomogram [22], and Ko et al. showed that osteocalcin adds independent predictive value beyond total ALP [19]. Ge et al. found that a comprehensive bone marker panel including osteocalcin, NTX, CTX, and TRAP-5b provided additional characterization of the bone turnover uncoupling that drives HBS [36]. Incorporating these markers could potentially improve model performance, particularly in the intermediate ALP range where clinical uncertainty is greatest [44]. Fifth, intraoperative PTH monitoring data—specifically the magnitude and rate of PTH decline—were not routinely collected at our center and therefore could not be included as features. Chai et al. identified percent PTH decay at skin closure as a key predictor in their model [29], suggesting that intraoperative data may capture real-time information about the acuteness of PTH withdrawal that preoperative variables alone cannot fully represent. Finally, parathyroid gland weight, which has been identified as a predictor in some nomograms [22], was not consistently recorded in our dataset and was therefore excluded.

In response to the aforementioned limitations, we have committed to a concrete external-validation roadmap. A multicenter prospective registry has been initiated with additional dialysis surgery centers, with a target of ≥200 surgical events over 36 months—sufficient power to externally validate the random forest model at the AUC = 0.85 lower bound and to retrain the model on a substantially larger and more heterogeneous cohort. Pre-specified validation endpoints include cross-validated AUC (target ≥ 0.85), composite bedside score calibration, and DCA at the 50% threshold. Until external validation is complete, the present model and bedside score should be regarded as internally validated, hypothesis-generating tools rather than ready for clinical adoption.

### 4.6. Future Directions

Several avenues for future research emerged from this work. External validation in independent, multicenter cohorts is the most pressing priority. Notably, Loftus et al. found that although 36.1% of AI-based surgical decision-support models presented a clinical implementation framework, none had assessed the efficacy of clinical implementation, with the absence of prospective validation identified as a principal barrier [39]. A multicenter registry incorporating diverse ethnic populations, varying surgical volumes, and different perioperative protocols would enable robust assessment of model transportability. Integration of intraoperative PTH kinetics and specialized bone turnover biomarkers (BSAP, P1NP, osteocalcin) as additional features could further refine the model, particularly for patients in the intermediate-risk ALP range of 250–300 U/L. The development of a web-based or mobile clinical decision support tool—embedding either the full ML model or the simplified composite risk score—could facilitate real-time preoperative risk stratification and guide individualized calcium replacement protocols. Finally, prospective studies comparing outcomes between standard care and ML-guided perioperative management would provide the highest level of evidence for the clinical utility of this approach.

## 5. Conclusions

This study demonstrates that ALP is so overwhelmingly dominant as a predictor of HBS following PTX for drug-refractory SHPT in dialysis patients that even a univariate ALP model achieved a cross-validated AUC of 0.958—numerically exceeding the best multi-feature random forest classifier (AUC = 0.933). While the multi-feature ML framework surpasses both published nomograms and the only prior dedicated ML model for this outcome, its primary contribution lies not in incremental discrimination over ALP alone, but in characterizing nonlinear dose–response relationships and interaction effects through SHAP analysis.

SHAP analysis confirmed preoperative ALP as the overwhelmingly dominant predictor, with a mean absolute SHAP value exceeding the next-ranked feature by more than sixfold, and revealed a sigmoid-shaped dose–response relationship with a clinically important inflection zone between 250–350 U/L. This nonlinear characterization refines the commonly used binary ALP > 300 U/L threshold by identifying an intermediate-risk zone (250–300 U/L) in which clinical decision-making should be individualized rather than guided by a single cutoff.

Beyond confirming ALP dominance, the ML and SHAP framework uncovered novel insights that conventional regression could not: baseline serum creatinine and preoperative calcium contribute to HBS risk through nonlinear interactions invisible to standard logistic models, and the choice of TPTX over SPTX amplifies ALP-mediated risk at lower ALP values—a previously uncharacterized effect modification with direct surgical planning implications. The finding that PTH adds no independent discriminative value beyond ALP challenges established clinical practice and suggests that risk assessment should be centered on bone turnover markers rather than PTH levels alone.

The SHAP-guided composite bedside risk score (range 0–9, AUC = 0.883) translates these ML-derived insights into a practical tool requiring only five routinely available preoperative variables (ALP value, severe bone pain, TPTX, PTH level, and creatinine > 10 mg/dL). Pending external prospective validation, the score may support preoperative risk stratification and help guide the intensity of perioperative calcium monitoring and supplementation, but should not yet be used as a stand-alone decision rule.

External validation in multicenter, ethnically diverse cohorts and prospective evaluation of ML-guided vs. standard perioperative management are essential next steps toward clinical implementation.

## Figures and Tables

**Table 4:** Pre-specified multi-criterion model-selection summary.

Model	AUC (Mean ± SD)	Brier (Mean ± SD)	F1 at Youden	NB at pt = 0.50
L1-Logistic (LASSO)	0.941 ± 0.041 ★	0.100 ± 0.038	0.836 ± 0.082	0.300
Random Forest	0.933 ± 0.065	0.111 ± 0.034	0.865 ± 0.081	0.344
L2-Logistic	0.930 ± 0.049	0.102 ± 0.036	0.847 ± 0.069	0.322
XGBoost	0.927 ± 0.074	0.098 ± 0.047 ★	0.877 ± 0.085 ★	0.356 ★
Neural Network	0.924 ± 0.062	0.117 ± 0.063	0.824 ± 0.103	0.311
SVM (RBF)	0.923 ± 0.063	0.114 ± 0.035	0.853 ± 0.079	0.322
Gradient Boosting	0.908 ± 0.069	0.153 ± 0.073	0.816 ± 0.085	0.267
KNN (k = 7)	0.791 ± 0.090	0.188 ± 0.038	0.569 ± 0.155	0.200
Univariate ALP (reference)	0.958 ± 0.041	—	—	0.367

★ marks the best value within each column among the eight multi-feature classifiers; the univariate-ALP logistic regression is shown for reference and is excluded from the multi-feature ranking. Brier and F1 values are not reported for the univariate-ALP reference row because only AUC was computed for the univariate model. Abbreviations: AUC = area under the receiver operating characteristic curve; SD = standard deviation; NB = net benefit; pt = threshold probability. Notes: Models are sorted by AUC. The univariate-ALP logistic regression is included for reference and is excluded from the multi-feature ranking. AUC quantifies overall discrimination across all decision thresholds. The Brier score quantifies probabilistic calibration, with lower values indicating better agreement between the predicted probabilities and observed outcomes. F1 at the Youden-optimal threshold balances sensitivity and precision at the operating point that maximizes the Youden index. Net benefit at pt = 0.50 quantifies clinical utility at a balanced operating threshold, reflecting the rate of true positives minus false positives weighted by the harm-to-benefit odds at that threshold.

**Table 5:** Side-by-side clinical-utility comparison of the multi-feature random forest, the univariate-ALP logistic regression, and the SHAP-guided composite bedside score.

Metric	Univariate ALP	Random Forest (24-Feature)	Composite Score (0–9)
Cross-validated AUC	0.958 ± 0.041	0.933 ± 0.065	0.883
Net benefit at pt = 0.30	0.376	0.343	0.321
Net benefit at pt = 0.40	0.352	0.348	0.307
Net benefit at pt = 0.50	0.367	0.344	0.289
Net benefit at pt = 0.60	0.278	0.356	0.261
Net benefit at pt = 0.70	0.289	0.344	0.215
Categorical NRI vs. ALP (overall)	—(reference)	−28.1%	not computed
Categorical NRI—events	—(reference)	−22.0%	not computed
Categorical NRI—non-events	—(reference)	−6.1%	not computed
Continuous NRI vs. ALP	—(reference)	−67.2%	not computed

Notes: Net benefit is reported at three threshold probabilities; categorical NRI uses the four risk tiers from Section 3.5.4; continuous NRI is shown for reference. AUC values are reproduced from Section 3.5.1 for ease of comparison. Net benefit is computed from out-of-fold predicted probabilities (5-fold CV × 10 repeats). The NRI of the composite score vs. ALP was not computed, since the score’s coarse integer scale (0–9) does not yield probabilities that are directly comparable to the continuous logistic-regression output. A negative NRI indicates that the candidate model reclassifies cases in the clinically less-helpful direction relative to the reference model.

## Data Availability

The original contributions presented in this study are included in the article. Further inquiries can be directed to the corresponding author.

## Abbreviations

The following abbreviations are used in this manuscript:

ALP	Alkaline phosphatase
AUC	Area under the curve
BSAP	Bone-specific alkaline phosphatase
CKD-MBD	Chronic kidney disease-Mineral and bone disorder
CTX	C-terminal telopeptide
CV	Cross-validation
EPV	Events-per-variable
ESRD	End-stage renal disease
HBS	Hungry bone syndrome
HPT	Hyperparathyroidism
iPTH	Intact parathyroid hormone
KNN	K-nearest neighbors
LASSO	Least absolute shrinkage and selection operator
ML	Machine learning
NPV	Negative predictive value
P1NP	Procollagen type I N-terminal propeptide
PDP	Partial dependence plot
PPV	Positive predictive value
PTH	Parathyroid hormone
PTX	Parathyroidectomy
RBF	Radial basis function
ROC	Receiver operating characteristic
SD	Standard deviation
SHAP	SHapley Additive exPlanations
SHPT	Secondary hyperparathyroidism
SPTX	Subtotal parathyroidectomy
SVM	Support vector machine
TPTX	Total parathyroidectomy
TRIPOD	Transparent Reporting of a Multivariable Prediction Model for Individual Prognosis or Diagnosis
USRDS	United States Renal Data System
VAS	Visual Analogue Scale

## Appendix A

**Table A1:** Complete list of 24 input features used in the primary machine-learning pipeline.

#	Feature	Type	Description/Units
**Raw Preoperative and Surgical Variables (** ***n*** ** = 19)**			
1	Age	Continuous	Years
2	Sex (Male)	Binary	1 = male, 0 = female
3	Dialysis vintage	Continuous	Years on renal replacement therapy
4	Preoperative serum calcium	Continuous	mg/dL, albumin-corrected
5	Preoperative intact PTH	Continuous	pg/mL
6	Baseline serum creatinine	Continuous	mg/dL
7	Alkaline phosphatase (ALP)	Continuous	U/L
8	Mild bone pain (VAS 1–3)	Binary	1 = present, 0 = absent
9	Moderate bone pain (VAS 4–6)	Binary	1 = present, 0 = absent
10	Severe bone pain (VAS 7–10)	Binary	1 = present, 0 = absent
11	Fatigability	Binary	1 = present, 0 = absent
12	Osteoporosis	Binary	1 = present, 0 = absent
13	Osteopenia	Binary	1 = present, 0 = absent
14	Pathological fracture	Binary	1 = present, 0 = absent
15	Number of enlarged glands on imaging	Discrete	Count (0–4)
16	Renal lithiasis	Binary	1 = present, 0 = absent
17	Preoperative calcium ≥ 11 mg/dL	Binary	From preop serum calcium
18	Surgical approach (TPTX vs. SPTX)	Binary	1 = TPTX, 0 = SPTX
19	Concomitant thymectomy	Binary	1 = performed, 0 = not performed
**Engineered Features—Binarized Thresholds (** ***n*** ** = 2)**			
20	PTH > 2000 pg/mL	Binary	From preoperative intact PTH
21	Glands ≤ 2	Binary	From number of enlarged glands
**Engineered Features—Interaction Terms (** ***n*** ** = 3)**			
22	ALP × severe bone pain	Continuous	Product of ALP and severe pain indicator
23	ALP × PTH/10,000	Continuous	Scaled product of ALP and PTH
24	PTH × TPTX/1000	Continuous	Scaled product of PTH and TPTX indicator

Notes: Features comprise 19 raw preoperative/surgical variables, 2 engineered binarized thresholds, and 3 engineered interaction terms. All 24 features were standardized (zero mean, unit variance) prior to model training, including binary features. Scaling divisors for interaction terms (#23, #24) were applied before standardization to maintain comparable feature magnitudes. The threshold for “preoperative calcium ≥ 11 mg/dL” uses ≥ rather than strict >, as patients with calcium = 11.0 mg/dL were coded as positive. The originally proposed binarized feature ‘ALP > 300 U/L’ was removed from the primary feature set because all 26 patients above this cut-point developed HBS in the dataset, producing a near-deterministic prediction rule rather than a learned pattern; sensitivity analysis with the 25-feature variant including this feature is reported in Appendix A Table A4.

**Table A2:** Case-by-case adjudication of all misclassified patients at the random forest Youden-optimal threshold of 0.647 (calcium + ALP/phosphate-inclusive HBS definition).

Case Type	Patient #	ALP (U/L)	Pain	Surgery	Preop Ca (mg/dL)	Postop Ca (mg/dL)	RF Probability
FP	11	284	Moderate	SPTX	9.6	8.2	0.701
FP	76	292	Mild	SPTX	9.2	8.6	0.667
FN	2	155	Moderate	SPTX	10.8	7.2	0.181
FN	7	248	Moderate	TPTX	10.1	6.8	0.271
FN	12	233	Severe	SPTX	9.9	6.8	0.278
FN	18	274	Moderate	SPTX	9.2	5.6	0.244
FN	27	282	Moderate	TPTX+AT	11.1	7.4	0.407
FN	59	256	Moderate	SPTX	10.1	6.5	0.114

Abbreviations: FP = false positive (predicted HBS, actual no HBS); FN = false negative (predicted no HBS, actual HBS). RF probability = out-of-fold predicted probability from 5-fold cross-validation × 10 repeats.

**Table A3:** Sensitivity analysis—original vs. calcium-only outcome.

Model	Original Outcome AUC	Calcium-Only Outcome AUC	ΔAUC
L1-Logistic (LASSO)	0.941 ± 0.041	0.887 ± 0.069	+0.054
Random Forest	0.933 ± 0.065	0.912 ± 0.080	+0.020
L2-Logistic	0.930 ± 0.049	0.861 ± 0.073	+0.068
XGBoost	0.927 ± 0.074	0.902 ± 0.075	+0.024
Neural Network	0.924 ± 0.062	0.882 ± 0.075	+0.042
SVM (RBF)	0.923 ± 0.063	0.903 ± 0.085	+0.020
Gradient Boosting	0.908 ± 0.069	0.882 ± 0.078	+0.027
KNN (k = 7)	0.791 ± 0.090	0.797 ± 0.100	−0.006
*Univariate ALP (logistic)*	0.958 ± 0.041	0.929 ± 0.064	+0.029
*Composite bedside score*	0.883	0.860	+0.024

Notes: Same 24-feature pipeline, identical hyperparameters, applied under two outcome definitions: original (postoperative ALP/phosphate-inclusive, *n*_HBS+ = 41) and a calcium-only proxy (HBS+ patients with postop calcium ≥ 7.6 mg/dL reclassified as HBS−, *n*_HBS+ = 39). Models sorted by descending AUC under the primary (original) outcome.

**Table A4:** Sensitivity analysis—pipeline with vs. without binarized ALP > 300 features.

Model	24-Feature (Primary)	25-Feature (with ALP > 300)	ΔAUC
L1-Logistic (LASSO)	0.941 ± 0.041	0.934 ± 0.048	−0.007
Random Forest	0.933 ± 0.065	0.934 ± 0.060	+0.001
L2-Logistic	0.930 ± 0.049	0.921 ± 0.057	−0.008
XGBoost	0.927 ± 0.074	0.926 ± 0.071	−0.000
Neural Network	0.924 ± 0.062	0.924 ± 0.051	+0.000
SVM (RBF)	0.923 ± 0.063	0.930 ± 0.067	+0.007
Gradient Boosting	0.908 ± 0.069	0.908 ± 0.070	−0.000
KNN (k = 7)	0.791 ± 0.090	0.867 ± 0.066	+0.076

Notes: Original outcome (*n* = 90, HBS+ = 41). Comparison of the primary 24-feature pipeline (binarized ALP > 300 excluded) against a 25-feature variant including the binarized feature. The empirical impact of this exclusion is negligible across all classifiers (ΔAUC < 0.01 for the seven non-KNN models).

**Table A5:** Random forest operating characteristics across candidate decision thresholds (out-of-fold predictions, 5-fold CV × 10 repeats, *n* = 90, HBS+ = 41).

Threshold	Sensitivity (%)	Specificity (%)	PPV (%)	NPV (%)	TP	FP	FN	TN	Scenario
0.20	95.1	51.0	61.9	92.6	39	24	2	25	Maximum-sensitivity
0.30	87.8	75.5	75.0	88.1	36	12	5	37	High-sensitivity
0.40	87.8	85.7	83.7	89.4	36	7	5	42	Balanced
0.50	85.4	91.8	89.7	88.2	35	4	6	45	Balanced (current default)
0.60	85.4	95.9	94.6	88.7	35	2	6	47	High-specificity
0.647	85.4	100.0	100.0	89.1	35	0	6	49	Youden-optimal
0.70	75.6	100.0	100.0	83.1	31	0	10	49	Maximum-specificity

Abbreviations: PPV = positive predictive value; NPV = negative predictive value; TP/FP/FN/TN = true/false positives/negatives. The 0.30 threshold is the lowest sensible operating point: thresholds below 0.30 produce only marginal additional sensitivity at the cost of substantial specificity loss. The 0.647 threshold maximizes the Youden index and corresponds to the operating point used for the confusion matrix in Figure 7A. All values are derived from out-of-fold predictions; in-sample estimates are not reported.

## References

[1] Cunningham J., Locatelli F., Rodriguez M. (2011). Secondary Hyperparathyroidism: Pathogenesis, Disease Progression, and Therapeutic Options. Clin. J. Am. Soc. Nephrol..

[2] Block G.A., Klassen P.S., Lazarus J.M., Ofsthun N., Lowrie E.G., Chertow G.M. (2004). Mineral Metabolism, Mortality, and Morbidity in Maintenance Hemodialysis. J. Am. Soc. Nephrol..

[3] Lau W.L., Obi Y., Kalantar-Zadeh K. (2018). Parathyroidectomy in the Management of Secondary Hyperparathyroidism. Clin. J. Am. Soc. Nephrol..

[4] Hiramitsu T., Hasegawa Y., Futamura K., Okada M., Goto N., Narumi S., Watarai Y., Tominaga Y., Ichimori T. (2023). Treatment for Secondary Hyperparathyroidism Focusing on Parathyroidectomy. Front. Endocrinol..

[5] National Kidney Foundation (2003). K/DOQI Clinical Practice Guidelines for Bone Metabolism and Disease in Chronic Kidney Disease. Am. J. Kidney Dis..

[6] (2017). Kidney Disease: Improving Global Outcomes (KDIGO) CKD-MBD Update Work Group. KDIGO 2017 Clinical Practice Guideline Update for the Diagnosis, Evaluation, Prevention, and Treatment of Chronic Kidney Disease–Mineral and Bone Disorder (CKD-MBD). Kidney Int. Suppl..

[7] Chen J., Zhou Q.-Y., Wang J.-D. (2015). Comparison Between Subtotal Parathyroidectomy and Total Parathyroidectomy with Autotransplantation for Secondary Hyperparathyroidism in Patients with Chronic Renal Failure: A Meta-Analysis. Horm. Metab. Res..

[8] Chen J., Jia X., Kong X., Wang Z., Cui M., Xu D. (2017). Total Parathyroidectomy with Autotransplantation versus Subtotal Parathyroidectomy for Renal Hyperparathyroidism: A Systematic Review and Meta-Analysis. Nephrology.

[9] Isaksson E., Ivarsson K., Akaberi S., Muth A., Prütz K.-G., Clyne N., Sterner G., Almquist M. (2019). Total versus Subtotal Parathyroidectomy for Secondary Hyperparathyroidism. Surgery.

[10] Schlosser K., Bartsch D.K., Diener M.K., Seiler C.M., Bruckner T., Nies C., Meyer M., Neudecker J., Goretzki P.E., Glockzin G. (2016). Total Parathyroidectomy with Routine Thymectomy and Autotransplantation versus Total Parathyroidectomy Alone for Secondary Hyperparathyroidism: Results of a Nonconfirmatory Multicenter Prospective Randomized Controlled Pilot Trial. Ann. Surg..

[11] Hou J., Shan H., Zhang Y., Deng X., Guo B., Kang J., Wu B., Fan Y. (2020). Network Meta-Analysis of Surgical Treatment for Secondary Hyperparathyroidism. Am. J. Otolaryngol..

[12] Cartwright C., Anastasopoulou C. (2025). Hungry Bone Syndrome. StatPearls.

[13] Jain N., Reilly R.F. (2017). Hungry Bone Syndrome. Curr. Opin. Nephrol. Hypertens..

[14] Mehta R., Rao K.N., Nagarkar N.M., Ghosh A., Sakale H. (2024). Hungry Bone Syndrome Following Parathyroidectomy: A Comprehensive Systematic Review of Risk Factors. Indian J. Surg..

[15] Kritmetapak K., Kongpetch S., Chotmongkol W., Raruenrom Y., Sangkhamanon S., Pongchaiyakul C. (2020). Incidence of and Risk Factors for Post-Parathyroidectomy Hungry Bone Syndrome in Patients with Secondary Hyperparathyroidism. Ren. Fail..

[16] Carsote M., Nistor C. (2023). Forestalling Hungry Bone Syndrome after Parathyroidectomy in Patients with Primary and Renal Hyperparathyroidism. Diagnostics.

[17] Ho L.-Y., Wong P.-N., Sin H.-K., Wong Y.-Y., Lo K.-C., Chan S.-F., Lo M.-W., Lo K.-Y., Mak S.-K., Wong A.K.-M. (2017). Risk Factors and Clinical Course of Hungry Bone Syndrome after Total Parathyroidectomy in Dialysis Patients with Secondary Hyperparathyroidism. BMC Nephrol..

[18] Coman A., Tarta C., Aiordachioae G.A., Goldis D., Utu D., Marian M., Dobrescu A., Buleu F., Olariu S. (2025). Predictors of Hungry Bone Syndrome and Reintervention After Subtotal versus Total Parathyroidectomy for Secondary Hyperparathyroidism in Dialysis Patients: A Single-Center Cohort. J. Clin. Med..

[19] Ko W.-C., Liu C.-L., Lee J.-J., Liu T.-P., Wu C.-J., Cheng S.-P. (2020). Osteocalcin Is an Independent Predictor for Hungry Bone Syndrome After Parathyroidectomy. World J. Surg..

[20] Witteveen J.E., van Thiel S., Romijn J.A., Hamdy N.A.T. (2013). Hungry Bone Syndrome: Still a Challenge in the Post-Operative Management of Primary Hyperparathyroidism: A Systematic Review of the Literature. Eur. J. Endocrinol..

[21] Amjad W., Ginzberg S.P., Passman J.E., Heintz J., Kelz R.R., Wachtel H. (2024). Predictive Risk Score for Postparathyroidectomy Hungry Bone Syndrome in Patients with Secondary Hyperparathyroidism. J. Clin. Endocrinol. Metab..

[22] Wang M., Chen B., Zou X., Wei T., Gong R., Zhu J., Li Z. (2020). A Nomogram to Predict Hungry Bone Syndrome After Parathyroidectomy in Patients with Secondary Hyperparathyroidism. J. Surg. Res..

[23] Gao D., Liu Y., Cui W., Lu X., Lou Y. (2024). A Nomogram Prediction Model for Hungry Bone Syndrome in Dialysis Patients with Secondary Hyperparathyroidism after Total Parathyroidectomy. Eur. J. Med. Res..

[24] Cao R., Jiang H., Liang G., Zhang W. (2024). Dynamic Nomogram for Predicting Hungry Bone Syndrome Before Parathyroidectomy. Endocrine.

[25] Muller O., Bauvin P., Bacoeur O., Michailos T., Bertoni M.-V., Demory C., Vankemmel A., Caiazzo R., Pattou F., Doyen J. (2024). Machine Learning-Based Algorithm for the Early Prediction of Postoperative Hypocalcemia Risk After Thyroidectomy. Ann. Surg..

[26] Seib C.D., Roose J.P., Hubbard A.E., Suh I. (2021). Ensemble Machine Learning for the Prediction of Patient-Level Outcomes Following Thyroidectomy. Am. J. Surg..

[27] Ramesh S., Vekaria S., Fisher J.C., Wright K., Underwood H., Prescott J., Allendorf J., Patel K.N., Suh I., Sum M. (2023). A Novel Risk Score to Predict Hungry Bone Syndrome After Parathyroidectomy for Renal Hyperparathyroidism. Endocr. Pract..

[28] Ding C., Guo Y., Mo Q., Ma J. (2022). Prediction Model of Postoperative Severe Hypocalcemia in Patients with Secondary Hyperparathyroidism Based on Logistic Regression and XGBoost Algorithm. Comput. Math. Methods Med..

[29] Chai Y., Yuan N., Yin J., Shen B., Sun L., Zhang L., Yin L., Wang X., Luo F., Luo C. (2025). Machine Learning-Based Predictive Model for Hungry Bone Syndrome Following Parathyroidectomy in Secondary Hyperparathyroidism. Front. Endocrinol..

[30] Lundberg S.M., Lee S.-I., Guyon I., Luxburg U.V., Bengio S., Wallach H., Fergus R., Vishwanathan S., Garnett R. (2017). A Unified Approach to Interpreting Model Predictions. Advances in Neural Information Processing Systems.

[31] Lundberg S.M., Nair B., Vavilala M.S., Horibe M., Eisses M.J., Adams T., Liston D.E., Low D.K.-W., Newman S.-F., Kim J. (2018). Explainable Machine-Learning Predictions for the Prevention of Hypoxaemia During Surgery. Nat. Biomed. Eng..

[32] Deng H., Eftekhari Z., Carlin C., Veerapong J., Fournier K.F., Johnston F.M., Dineen S.P., Powers B.D., Hendrix R., Lambert L.A. (2022). Development and Validation of an Explainable Machine Learning Model for Major Complications After Cytoreductive Surgery. JAMA Netw. Open.

[33] Collins G.S., Moons K.G.M., Dhiman P., Riley R.D., Beam A.L., Van Calster B., Ghassemi M., Liu X., Reitsma J.B., van Smeden M. (2024). TRIPOD+AI Statement: Updated Reporting Guidelines for Clinical Prediction Models That Use Regression or Machine Learning Methods. BMJ.

[34] Christodoulou E., Ma J., Collins G.S., Steyerberg E.W., Verbakel J.Y., Van Calster B. (2019). A Systematic Review Shows No Performance Benefit of Machine Learning over Logistic Regression for Clinical Prediction Models. J. Clin. Epidemiol..

[35] Coman A., Tarta C., Marian M., Popa D.I., Olariu S., Rosu M., Utu D., Buleu F., Macovei-Oprescu A.M., Novacescu D. (2025). Hungry Bone Syndrome After Parathyroidectomy for Secondary Hyperparathyroidism: Pathogenesis and Contemporary Clinical Considerations. J. Clin. Med..

[36] Ge Y., Yang G., Wang N., Zha X., Yu X., Mao H., Sun B., Zeng M., Zhang B., Xing C. (2019). Bone Metabolism Markers and Hungry Bone Syndrome After Parathyroidectomy in Dialysis Patients with Secondary Hyperparathyroidism. Int. Urol. Nephrol..

[37] Peng X., Xia X., Li Z., Cheng F., Zhu X. (2022). Factors Influencing the Development of Bone Starvation Syndrome After Total Parathyroidectomy in Patients with Renal Hyperparathyroidism. Front. Surg..

[38] Tai Y.L., Shen H.Y., Nai W.H., Fu J.F., Wang I.K., Huang C.C., Weng C.H., Lee C.C., Huang W.H., Yang H.Y. (2023). Hungry Bone Syndrome After Parathyroid Surgery. Hemodial. Int..

[39] Loftus T.J., Altieri M.S., Balch J.A., Abbott K.L., Choi J., Marwaha J.S., Hashimoto D.A., Brat G.A., Raftopoulos Y., Evans H.L. (2023). Artificial Intelligence-Enabled Decision Support in Surgery: State-of-the-Art and Future Directions. Ann. Surg..

[40] Hong N., Lee J., Kim H.W., Jeong J.J., Huh K.H., Rhee Y. (2022). Machine Learning–Derived Integer-Based Score and Prediction of Tertiary Hyperparathyroidism among Kidney Transplant Recipients. Clin. J. Am. Soc. Nephrol..

[41] Xie F., Chakraborty B., Ong M.E.H., Goldstein B.A., Liu N. (2020). AutoScore: A Machine Learning–Based Automatic Clinical Score Generator and Its Application to Mortality Prediction Using Electronic Health Records. JMIR Med. Inform..

[42] Oh M.Y., Kim H.S., Jung Y.M., Lee H.C., Lee S.B., Lee S.M. (2025). Machine Learning–Based Explainable Automated Nonlinear Computation Scoring System for Health Score and an Application for Prediction of Perioperative Stroke: Retrospective Study. J. Med. Internet Res..

[43] Wong J., Fu W.H., Lim E.L.A., Ng C.F.J., Choong H.L. (2020). Hungry Bone Syndrome After Parathyroidectomy in End-Stage Renal Disease Patients: Review of an Alkaline Phosphatase-Based Treatment Protocol. Int. Urol. Nephrol..

[44] Coman A., Tarta C., Isaic A., Marian M., Olariu S., Ardelean A., Macovei-Oprescu A.M., Roland F., Pupca G.N., Latcu S. (2025). Predictors of Hungry Bone Syndrome After Parathyroidectomy in Secondary Hyperparathyroidism: A Narrative Review of Bone Turnover Biomarkers and Risk Prediction Tools. J. Clin. Med..

